# Phase II Study of *Pseudomonas aeruginosa*-Mannose-Sensitive Hemagglutinin in Combination with Capecitabine for Her-2–Negative Metastatic Breast Cancer Pretreated with Anthracycline and Taxane

**DOI:** 10.1371/journal.pone.0118607

**Published:** 2015-03-13

**Authors:** Fangfang Lv, Jun Cao, Zhebin Liu, Zhonghua Wang, Jian Zhang, Sheng Zhang, Leiping Wang, Xinmin Zhao, Zhimin Shao, Biyun Wang, Xichun Hu

**Affiliations:** 1 Department of Medical Oncology, Fudan University Shanghai Cancer Center, Shanghai, China, Department of Oncology, Shanghai Medical College, Fudan University, Shanghai, China; 2 Department of Breast Surgery, Fudan University Shanghai Cancer Center, Shanghai, China, Department of Oncology, Shanghai Medical College, Fudan University, Shanghai, China; Stavanger University Hospital, NORWAY

## Abstract

**Purpose:**

Metastatic breast cancer (MBC) remains an incurable disease despite major therapeutic advances. *Pseudomonas aeruginosa*–mannose-sensitive hemagglutinin (PA-MSHA) has been established to have anti-proliferative effects against breast cancer cells in preclinical experiments, and is indicated for treatment of cancer in China. We performed a phase II trial combining PA-MSHA with capecitabine in patients with heavily pretreated MBC.

**Methods:**

Eligibility criteria included human epidermal growth factor receptor 2–negative MBC, prior therapy with anthracyclines and taxanes, at least one prior chemotherapy regimen for metastatic disease or early relapse after a taxane plus anthracycline adjuvant regimen, and adequate organ function and performance status. PA-MSHA 1 mg was administered subcutaneously every other day and capecitabine 1000 mg/m^2^ orally twice a day for 2 weeks on, 1 week off. The primary end point was progression-free survival.

**Results:**

A total of 97 patients were enrolled. Median progression-free survival (PFS) was 4.0 months [95 % confidence interval (CI) 3.0–4.9], which was not significantly different from that in historical controls. However, median PFS was significantly longer (8.2 months; 95 % CI 6.7–9.7) in 24 patients with moderate immune-related adverse events (irAEs) such as fever or skin induration at the injection site than in those with no or mild irAEs (3.1 months, 95 % CI 2.5–3.6; *p* = 0.003). Overall survival was also improved in these patients (25.4 vs. 16.4 months; *p* = 0.044). PA-MSHA has a good safety profile, with only 6 patients (6.2 %) discontinuing treatment. PA-MSHA did not increase capecitabine-related toxicities such as hand-foot syndrome, nausea, and vomiting.

**Conclusion:**

Adding PA-MSHA to capecitabine has a good safety profile in patients with heavily pre-treated MBC, although benefit from this regimen might occur only in patients with moderate PA-MSHA–related adverse events.

**Trial Registration:**

ClinicalTrials.gov NCT01380808

## Introduction

Despite advances in conventional cytotoxic chemotherapy and targeted therapy, metastatic breast cancer (MBC) remains, with few exceptions, an incurable disease, and effective new treatments are needed. Salvage chemotherapy produces a modest benefit for breast cancer that has progressed after prior anthracycline and taxane therapy. A widely used drug in this setting is capecitabine [1], which can be used conveniently with mild toxicities. Single-agent capecitabine treatment for taxane- and anthracycline-resistant disease has been associated with a response rate of 15–28% [2–4] in several phase II clinical trials. Progression-free survival (PFS) is around 4 months and median overall survival (OS) is about 13.5 months [5]. Several drugs have been investigated for use in combination with capecitabine to improve survival in patients who have been pretreated with anthracycline and taxane. Disappointingly, only a few target agents such as tratuzumab and lapatinib [6] have been recommended for combination therapy with capecitabine for human epidermal growth factor receptor 2 (Her-2)–positive breast cancer to improve outcomes, and other drugs, including sunitinib [7] and bevacizumab [8], have failed to improve outcomes in Her-2–negative breast cancer. Although ixabepilone [9] has been shown to increase the overall response rate (ORR) and PFS when combined with capecitabine, it is also associated with markedly increased toxicities.

Heat-killed *Pseudomonas aeruginosa*, a gram-negative bacterium, has been successfully used for anti-infection and has even been used in anti-tumor therapies as an immune activator. *P*. *aeruginosa*–mannose-sensitive hemagglutinin (PA-MSHA) is a genetically established, engineered *P*. *aeruginosa* strain characterized by the expression of mannose-sensitive type 1 fimbriae on its surface. PA-MSHA has been approved by the State Food And Drug Administration for complementary cancer treatment in China since 1998 because of its anticarcinogenic activities against human gastric cancer cells and hepatocarcinoma cells. In our earlier study, we found PA-MSHA also had a cytotoxic effect in human breast cancer cell lines and might have antiproliferative effects against breast cancer cells by inducing apoptosis mediated via modulating caspase family proteins and affecting cell cycle regulation machinery [10]. After that, we confirmed the antitumor effect of PA-MSHA both in breast cancer cell lines MDA-MB-231HM and MDA-MB-468 and in mice bearing tumor xenografts [11]. Both of these two breast cancer cell lines are HER-1 positive and HER-2 negative. PA-MSHA-treated cells have significantly lower expression levels of the oncogenes vascular endothelial growth factor, matrix metalloproteinase, and cathepsin-D, and a significantly higher expression level of tumor suppressor gene E-cadherin, which suggests that PA-MSHA can inhibit the invasive ability of the cell lines MDA-MB-231HM and MDA-MB-468. The incidence of lung metastasis was also lower in PA-MSHA-treated nude mice models.

Additionally, PA-MSHA might activate the immune system thereby enabling cancer regression. Toll-like receptor [12] located on the surface of immune cells such as dendritic cells can be activated by MSHA fimbriae. Thus, dendritic cells may be induced to mature and further activate cytotoxic T lymphocytes and natural killer cells, which might induce the antitumor specific and non-specific immune reactions.

In a phase II clinical trial with a small sample size [13], PA-MSHA was investigated as neoadjuvant chemotherapy for breast cancer. ORR and pathological complete remission (CR) were both improved with the addition of PA-MSHA to the cytotoxic drugs without adding severe toxicities.

On the basis of these encouraging preclinical and clinical data, we performed a single arm, phase II clinical trial using PA-MSHA combined with capecitabine as salvage treatment for Her-2–negative MBC patients pretreated with anthracyclines and taxanes.

## Methods

The protocol for this trial and supporting TREND checklist are available as supporting information; see S1 Checklist and S1 and S2 Protocols.

### Patients

Women aged between 18 and 70 years with histologically confirmed Her-2–negative MBC were eligible. Patients were required to have an Eastern Cooperative Oncology Group performance status of no more than 2 and evidence of adequate organ function. Patients must have had prior anthracycline and taxane treatment in the early or advanced disease setting. Either measurable or evaluable bone-only metastatic disease was permitted according to the Response Evaluation Criteria in Solid Tumors (RECIST) version 1.1. Prior capecitabine-containing therapy was permitted if the disease had responded to the drug previously and progressed at least 4 months after drug discontinuation. Patients with known dihydropyrimidine dehydrogenase deficiency or known hypersensitivity to PA-MSHA were excluded.

The study was conducted in accordance with the International Conference on Harmonisation Good Clinical Practice guidelines, the Declaration of Helsinki, and applicable local regulatory requirements and laws. Study procedures were approved by the institutional review board of the Fudan University Shanghai Cancer Center. Written informed consent was obtained from all patients.

### Study design and treatments

This was a single-arm phase II study. The primary end point was PFS (defined as time from the date of enrollment to first documented tumor progression or death during study as a result of any cause, whichever occurred first). Secondary end points included ORR (defined as the proportion of patients with CR or partial remission [PR] during treatment at the time gaining best response), OS (defined as the time from the date of enrollment until the date of death, regardless of the cause of death), and safety profile and immune indices. Patients who were alive at the time of the final analysis will be censored at the date of the last follow-up assessment. Patients received capecitabine orally at a starting dose of 2,000 mg/m^2^/day (1,000 mg/m^2^ twice daily) on days 1 to 14 of a 3-week cycle plus PA-MSHA subcutaneously at a dose of 1 ml every other day (0.5 ml on the first day). Treatment was continued until disease progression, unacceptable toxicity, or consent withdrawal. If PA-MSHA or capecitabine was discontinued for reasons other than progression, patients could continue receiving the other drug until progression. Patients were monitored for toxicity. The PA-MSHA dose could be reduced to 1 ml every 3 days based on individual tolerability. Capecitabine dose adjustments were performed according to the approved label.

### Study procedures

Tumor assessment was performed by the investigators using computed tomography, spiral computed tomography, or magnetic resonance imaging at baseline and every two cycles until disease progression or death occurred, according to the RECIST. Safety was assessed at each cycle; adverse events were graded using the National Cancer Institute Common Terminology Criteria for Adverse Events version 3.0. Adverse event data were collected up to 28 days after the last dose of study medication.

### Statistical analysis

The median PFS for patients receiving capecitabine was assumed to be 4.2 months [9], Therefore, the null hypothesis of PFS of 4.2 months or lower was tested against the alternative hypothesis of a true PFS of 5.8 months [9] or higher with addition of PA-MSHA. The sample size was calculated as 88 patients, with a two-sided alpha-level of 0.05 (18 months enrollment duration, 6 months follow up duration after enrollment). The statistic test power was 80%. With an expected discontinuation rate of 10%, 96 or more patients were to be enrolled. At least 70% PFS events were needed before follow-up ended.

Descriptive statistics were used to summarize patient characteristics and treatment administration and compliance. Time-to-event end points were summarized using the Kaplan-Meier method (SPSS 16.0). Log-rank test was used to test time-to-event subgroup differences. ORR difference between subgroups was analysed using Chi-square test. The main statistic was efficacy in both the full analysis set (FAS) and the per protocol population. The safety data set included all patients who received at least one dose of study medication, which was evaluated for compliance and safety.

## Results

### Patients and treatment exposure

**Fig 1:**
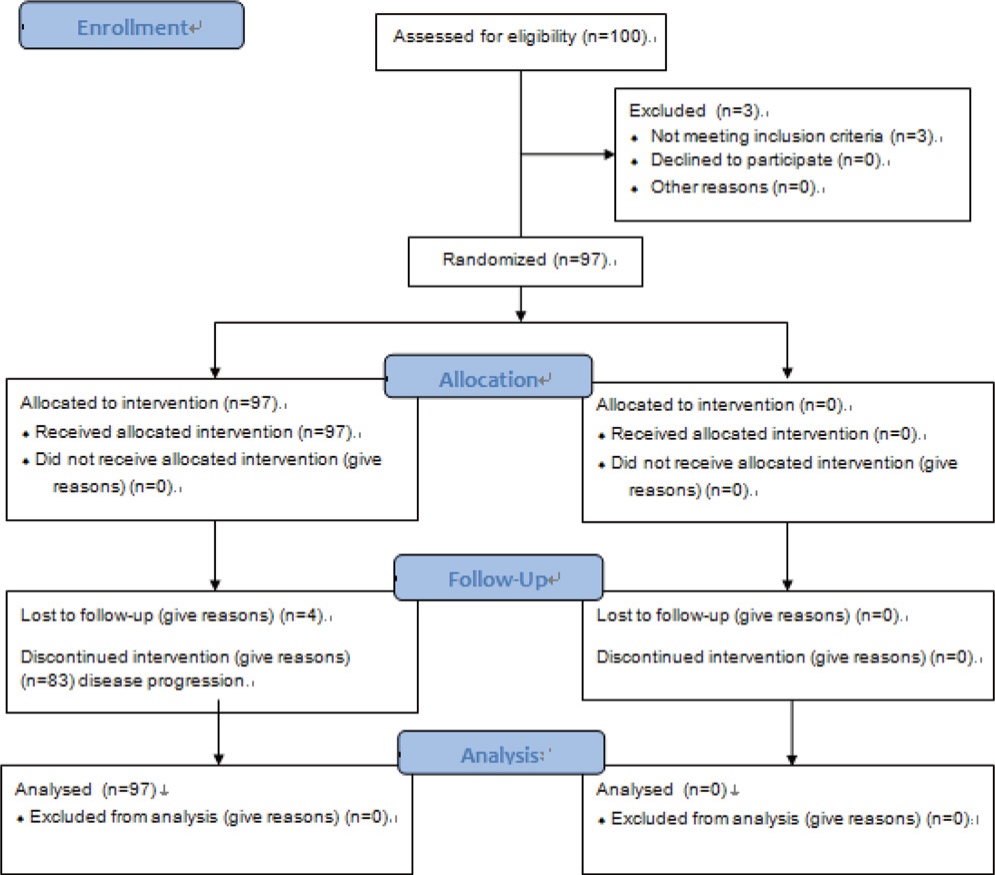
Consort flow chart of this trial.

From May 2011 to Feb 2013, 100 women signed the informed consent form and 97 were eligible for study entry and formed the FAS population (Fig. 1). All eligible patients received at least one dose of capecitabine and PA-MSHA and were included in the safety analyses. Demographic and baseline disease characteristics of the FAS population are listed in Table 1.

**Table 1:** Patient characteristics (*n* = 97).

Characteristic	Whole population (*n* = 97) Number (%)
Median age, years (range)	52 (24–70)
Amenorrhea	
Premenopausal	28 (28.9)
Postmenopausal	69 (71.1)
Advanced or metastatic	
De novo metastatic	2 (2.1)
Metastatic	95 (97.9)
No. of metastatic sites	
1	13 (13.4)
2	30 (30.9)
≥3	54 (55.7)
Metastatic sites	
Visceral	76 (78.4)
Lung	53 (54.6)
Liver	39 (40.2)
Non-visceral	21 (21.6)
Bone	51 (52.6)
ER status	
Positive	74 (76.3)
Negative	23 (23.7)
PR status	
Positive	57 (58.8)
Negative	40 (41.2%)
Prior chemotherapy regimens	
Adjuvant/neoadjuvant only	24 (24.7)
One for advanced breast cancer	53 (54.6)
≥2 for advanced breast cancer	20 (20.6)
Subgroup	
Triple negative	20 (20.6)
Luminal type	77 (79.4)

*ER* estrogen receptor, *PR* progesterone receptor

At data cutoff (October 2013), four patients were lost follow-up with no survival data. A total of 83 patients had fulfilled the study protocol and another 10 patients are still receiving study drugs. The median duration of follow-up was 22.0 months. Twelve patients had dose reduction, of whom 8 patients had capecitabine dose reduction due to grade 3 hand-foot syndrome (HFS) or grade 3 hepatic toxicity (7 and 1, respectively), and 6 patients had PA-MSHA dose reduction due to grade 2 immune-related adverse events (irAEs; 5 for skin induration at the injection site, 1 for fever). Two patients had dose reduction of both drugs.

### Efficacy

**Fig 2:**
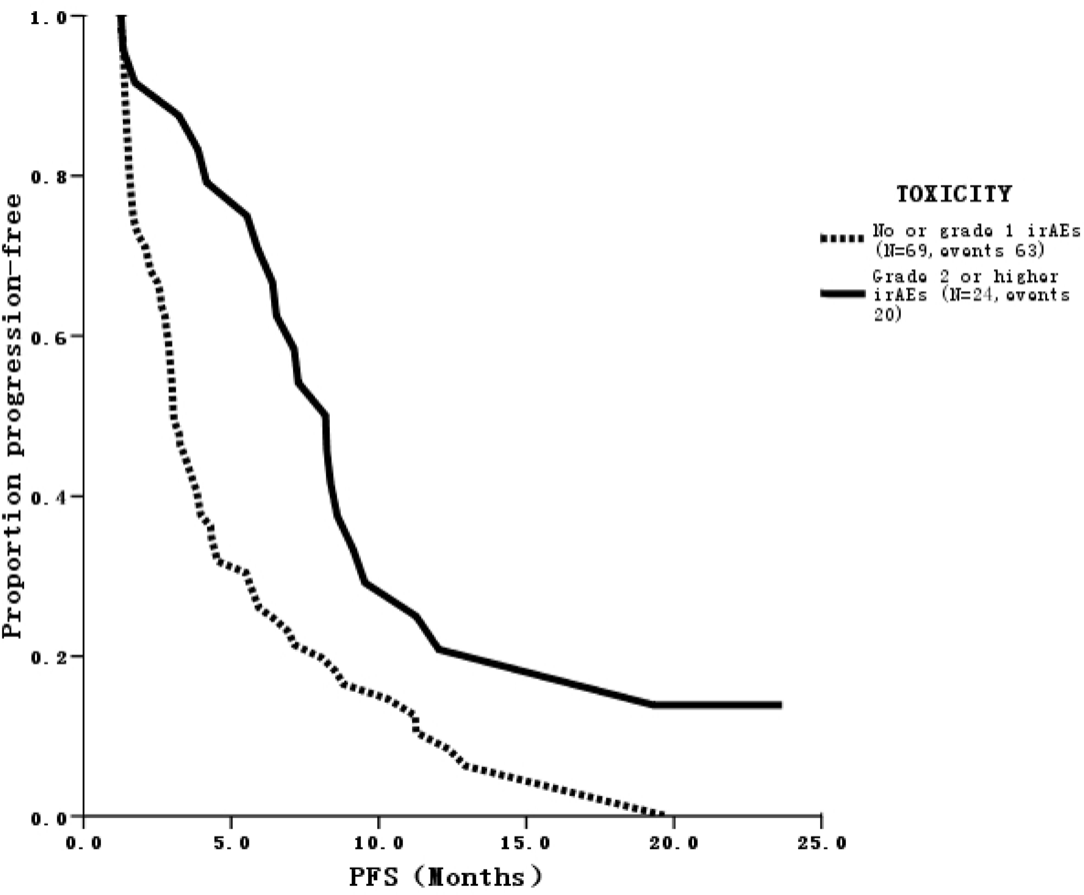
PFS curve of patients with different grades of irAEs. Patients with grade 2 or higher irAEs (N = 24, events 20) had longer PFS than those who had no or grade 1 irAEs (N = 69, events 63) (*p* = 0.003).

**Fig 3:**
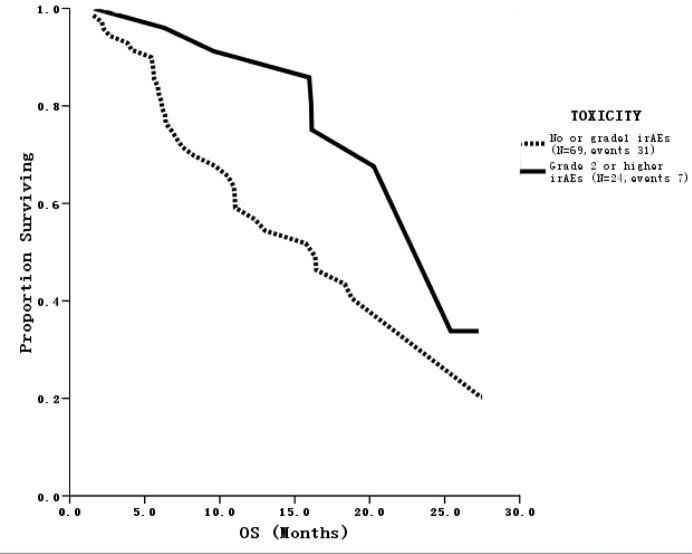
OS curve of patients with different grades of irAEs. Patients with grade 2 or higher irAEs (N = 24, events 7) had longer OS than those who had no or grade 1 irAEs (N = 69, events 31) (*p* = 0.044).

After a median follow-up of 22 months, 83 (85.6%) patients had disease progression and 38 (39.2%) patients had died. Twenty-one patients achieved PR and no patient had CR, for an ORR of 21.6% (Table 2). Among the 21 patients who responded, 11 achieved PR in cycle 2, eight in cycle 4 and two at the end of cycle 6. Median PFS was 4.0 months [95% confidence interval (CI), 3.0–4.9], which was not significantly different from the historical control and the literature [9]. However, median PFS was significantly longer (8.2 months) in 24 patients with grade 2 or higher irAEs such as fever or skin induration at the injection site than in those who had no or grade 1 irAEs (3.1 months; *p* = 0.003) (Fig. 2). This phenomenon was observed regardless of the presence of visceral metastases or estrogen receptor status. In addition, ORR was higher in this population (36% vs. 16.7%; *p* = 0.043). Furthermore, median OS was also significantly improved in these patients (25.4 months vs. 16.4 months; *p* = 0.044) (Fig. 3).

**Table 2:** Efficacy results: patients with grade 2 or higher irAEs had better ORR than those with no or grade 1 toxicities.

	Patients with no or grade 1 irAEs (*n* = 72) Number (%)	Patients with grade 2 or higher irAEs (*n* = 25) Number (%)	Whole population (*n* = 97) Number (%)
CR	0	0	0
PR	12 (16.7)	9 (36.0)	21 (21.6)
SD	33 (45.8)	12 (48.0)	45 (46.4)
PD	21 (29.2)	2 (8.0)	23 (23.7)
UK	6 (8.3)	2 (8.0)	8 (8.2)

*CR* complete remission, *irAE* immune-related adverse events, *PD* progressive disease, *PR* partial remission, *SD* stable disease, *UK* not known

*irAE* immune-related adverse events, *PFS* progression-free survival

*irAE* immune-related adverse events, *OS* overall survival

ORR was also analyzed in subgroups for different ER/PR status and luminal subtype or triple-negative subtype. ORR was higher in luminal subtype [23.4% (18/77) v.s. 15% (3/20), p = 0.418], ER positive subgroup [24.3% (18/74) v.s. 13.0% (3/23), p = 0.251] and PR positive subgroup [26.3% (15/57) v.s. 15% (6/40), p = 0.226]. However, the difference did not have statistical significance.

### Safety

All patients who received at least one dose of PA-MSHA and capecitabine were analyzed for safety. The toxicity profiles are summarized in Table 3. PA-MSHA did not increase the hematologic toxicities of capecitabine. The incidence of the most common grade 3/4 hematologic toxicities such as neutropenia, leukopenia, and thrombocytopenia were all less than 5%. The most common grade 3/4 non-hematologic toxicity was HFS (5.2%). Other grade 3/4 non-hematologic toxicities such as fever, mucositis, and rash were uncommon. The toxicities caused by PA-MSHA mainly included fever, skin induration at the injection site, and rash. These toxicities were all mild or moderate except for 2 patients who developed grade 3 rash and 2 who developed grade 3 fever. PA-MSHA toxicities were manageable and only 6 patients (6.2%) discontinued PA-MSHA due to toxicity.

**Table 3:** Main toxicities of PA-MSHA combined with capecitabine (*n* = 97).

Toxicity	All grades Number (%)	Grade ¾ Number (%)
Hematologic		
Neutropenia	23 (23.7)	3 (3.1)
Leukopenia	24 (24.7)	3 (3.1)
Thrombocytopenia	5 (5.2)	2 (2.1)
Anemia	15 (15.5)	1 (1.0)
Non-hematologic		
Hand-foot syndrome	31 (32.0)	5 (5.2)
Rash	5 (5.2)	2 (2.1)
Fever	29 (29.9)	2 (2.1)
Skin induration at the injection site	43 (44.3)	0
Fatigue	12 (12.4)	0
Nausea	11 (11.3)	0
Vomiting	4 (4.1)	0
Diarrhea	4 (4.1)	0
Stomatitis	4 (4.1)	1 (1.0)
Liver dysfunction	6 (6.2)	0
Abdominal pain	6 (6.2)	0
Stomach discomfort	2 (2.1)	0
Loss of appetite	16 (16.5)	0
Hyperpigmentation	21 (21.6)	0

## Discussion

This prospective single-arm phase II trial evaluated the efficacy of PA-MSHA, in combination with capecitabine, in the treatment of anthracycline- and taxane-pretreated Her-2–negative MBC patients. Studies have already indicated that PA-MSHA can increase the antigen presenting function by activating the proliferation and differentiation of dendritic cells [14]. Thus, PA-MSHA may increase the number and proportion of T cells and stimulate cell-mediated immunity. On the other hand, PA-MSHA has been shown to induce cytotoxic effects against estrogen receptor—and progesterone receptor–negative breast cancer cells lines [11] and in breast cancer xenografts. However, this has not been validated in the clinical setting. To our knowledge, this is the first clinical study to test the anti-cancer efficacy and safety profile of PA-MSHA in Her-2–negative MBC patients.

Disappointingly, the addition of PA-MSHA to capecitabine did not improve the outcomes in the whole patient population. PFS and ORR were also not increased compared with historic controls. It seems that PA-MSHA did not increase the anticancer effects of capecitabine in MBC. However, the response criteria for cytotoxic therapy are not suitable for immune therapy in our opinion. We noticed that the 4.0-month median PFS in our study was mainly due to 20 patients who had early disease progression at the end of cycle 2. Only half of the responders had their best response at cycle 2, and two patients did not achieve PR until the end of cycle 6. Data show that the appearance of measurable antitumor activity may take longer for immune therapies than for cytotoxic therapies [15]. Some responses to immune therapies may occur even after conventional radiological progressive disease (PD) has occurred. It is recommended that discontinuation of immune therapy may not be appropriate in some patients unless PD has been confirmed. ‘Clinically insignificant’ PD (e.g. small new lesions in the presence of other responsive lesions) is thought not really to be PD in this setting and durable stable disease may represent antitumor activity [16]. The response to PA-MSHA in our study might be similar to that of other immune therapies, although our study was not purely an immune therapy study, given the addition of capecitabine. We noticed a relatively slow response in our study compared with other chemotherapy regimens in the literature. Some patients discontinued the study because of conventional radiological PD according to the RECIST; there may have been some patients who could potentially have benefitted from longer administration of the study regimen. In summary, the different evaluation systems might be a reason why PFS was not improved in our study. Outcomes might have been better if immune-related response criteria [16] were used.

On the other hand, the addition of PA-MSHA to capecitabine did not increase the toxicity and was well tolerated. Only immune reaction–related toxicities such as fever, skin indurations, and rash occurred more frequently than in historical controls. This phenomenon can be due to the immune activating effects of PA-MSHA. Interestingly, the efficacy in patients who had grade 2 or higher irAEs was significantly improved when compared with the whole population. The ORR was much higher (36%) in these patients. The improvement in PFS in these patients was 5 months (8.2 months vs. 3.1months; *p* = 0.003). Importantly, OS was also significantly prolonged in this population (25.4 months vs. 16.4 months; *p* = 0.044). It seems that patients who had an obvious immune reaction also had a better response to PA-MSHA. Although the comparison of grade 2 or higher irAEs ORR/OS were post-hoc tests and were not pre-specified, we did see this phenomenon and presume fever and skin indurations might be predictors of response to PA-MSHA. All of the irAEs were tolerated and easily managed. Although we also collected blood samples for cytokine detection, the relationship between serum cytokine level and response is not discussed in this paper.

The addition of PA-MSHA did not increase hematologic or non-hematologic toxicities of capecitabine in our study. On the contrary, the incidences of adverse events commonly associated with capecitabine such as hand-foot syndrome, nausea and vomiting, and diarrhea were all reduced by the addition of PA-MSHA. The incidences of leukopenia and neutropenia were also lower compared with the historical controls and literature reports, and these toxicities were manageable. No febrile neutropenia occurred in the whole population. The dose reduction of capecitabine was less common than in previous phase III studies [7–9], which may be due to the enhancement of the patients’ immune systems. Similar results were seen in several small sample phase II clinical trials [13]. In these neo-adjuvant settings, the addition of PA-MSHA decreased the toxicities induced by chemotherapy in breast cancer patients. However, randomized trials are warranted to confirm these toxicity results.

Our study demonstrates that PA-MSHA in combination with capecitabine possesses superior clinical efficacy in patients with grade 2 or higher irAEs for MBC that has progressed after multiple prior treatments, including anthracyclines and taxanes. Although no significant improvement was seen in the whole population, we presume benefit would be seen with immune-related response criteria. A randomized controlled clinical trial might be needed to confirm the result.

## Supporting Information

S1 Checklist Trend Checklist. (PDF)

S1 Protocol Protocol Chinese. (DOC)

S2 Protocol Protocol English. (DOC)
