# Supplementary material for: Phase II Study of Pseudomonas aeruginosa-Mannose-Sensitive Hemagglutinin in Combination with Capecitabine for Her-2–Negative Metastatic Breast Cancer Pretreated with Anthracycline and Taxane
Source: PLoS One. 2015 Mar 13;10(3):e0118607. doi: 10.1371/journal.pone.0118607 (PMC4359133; doi:10.1371/journal.pone.0118607)
Supplement: S1 Protocol — (DOC) [file pone.0118607.s002.doc]

**希罗达联合万特普安解救治疗HER-2阴性的**

**转移性乳腺癌的II期临床研究**

**临床研究方案**

**（方案编号：）**

|  |  |
| --- | --- |
|  |  |

**方案制定日期：2011/02/01**

**版本号：1.0**

**摘要**

| 研究题目 | 希罗达联合万特普安解救治疗HER-2阴性的转移性乳腺癌的II期临床研究 |
| --- | --- |
| 研究单位 | 复旦大学附属肿瘤医院 |
| 研究目的 | 评价希罗达联合万特普安解救治疗转移性乳腺癌的有效性和安全性。 |
| 研究设计 | 单臂、开放、单中心II期临床研究 |
| 受试者 | 一线或二线治疗失败的转移性乳腺癌患者96例 |
| 研究分组 | 入组96例：单组希罗达＋万特普安 |
| 研究方法 | 研究方案：希罗达1000 mg/m^2^ Bid，PO，d1-14，21天为一个周期，用至疾病进展或发生不可耐受的毒性。口服希罗达同时，从希罗达开始第一天起皮下注射万特普安（建议腹部和健侧上臂皮下注射），隔天一次连续给药，直至疾病进展或发生不可耐受的毒性。 |
| 主要终点 | 无疾病进展生存期（PFS） |
| 次要终点 | 疾病缓解率（ORR）、总生存期（OS）、安全性、肿瘤转移相关指标、免疫学指标。 |
| 入选标准 | 1) 女性，年龄18~70岁；  2) 组织病理学确诊为浸润性乳腺癌，并发生转移不适合行局部治疗的患者；  3) 病理组织学检查确定HER-2表达呈阴性的患者（免疫组化检测HER-2为-或+，若HER-2为++，则需进行FISH检测，结果为阴性）；  4) 经蒽环类和/或紫杉类方案治疗后进展，适合行希罗达治疗的患者；   1. 既往未用过含希罗达的方案治疗或含希罗达方案治疗有效，停化疗4月以上疾病发生进展的患者； 2. Karnofsky评分≥70；ECOG评分0~2；   7) 心脏、肝脏、肾脏及骨髓造血功能良好；  8) 实验室检查符合WBC≥4×10^9^/L；Hb≥90 g/L；plt≥100×10^9^/L；  9) 患者自愿参加，签署知情同意书。 |

**一、背景资料**

**1. 转移性乳腺癌的治疗现状**

乳腺癌是女性最常见的恶性肿瘤，全球每年约有100万新发乳腺癌患者，每年有超过41万患者死于乳腺癌。近年我国乳腺癌发病率亦快速上升，在2000年，中国女性的乳腺癌年龄标化发病率为19.9/10万，已经跃居我国女性恶性肿瘤第一位，尽管乳腺癌的诊断和治疗有了较大进展，但由于肿瘤本身的生物学行为特点以及乳腺癌的早期诊断率仍较低等因素，大多数乳腺癌患者最终仍出现复发转移。对于复发转移性乳腺癌，目前认为是不可治愈性疾病，中位生存期仅2-3年*。*转移性乳腺癌治疗的主要目的是延长生存期、减少疾病相关症状以及提高患者的生活质量。因此，探索新的抗肿瘤药物和治疗方案，延长患者的生存期、降低治疗的毒副反应、提高患者的生活质量，就尤为重要。

最近研究显示利用基因芯片技术（cDNA microarray）将侵袭性乳腺癌分为若干基本亚型，包括luminal A型、luminal B型及激素受体阴性的亚型，即HER2阳性型及 basal-like亚型。在常规的临床治疗中，根据患者是否过度表达激素受体（雌激素受体ER和/或孕激素受体 PgR）和/或人类表皮生长因子受体2蛋白（HER-2）进行分类。对激素受体阳性的肿瘤，内分泌治疗由于其疗效较好、便于使用以及毒性极低而成为通常的选择。对HER2阳性型常选择以Herceptin为基础的治疗方案。对三阴性或虽激素受体阳性但内分泌治疗耐药的患者，细胞毒性化疗是标准的治疗。单药和联合治疗方案都是治疗转移性乳腺癌有效的选择。尽管联合治疗在疾病缓解率和疾病进展时间上有所提高，但在生存上基本没有获益，并且多种细胞毒药物同时给药明显增加了毒性。

在转移性乳腺癌的治疗中常用的化疗药物包括蒽环类（如多柔比星、表柔比星）、紫杉烷类（如多西他赛、紫杉醇）、烷化剂（如环磷酰胺）、卡培他滨（希罗达）、长春瑞滨和吉西他滨^[1]^。蒽环类被认为是乳腺癌最有效的治疗药物之一，通常应用于初始治疗。紫杉烷类也已被认为是治疗转移性乳腺癌非常有效的一类药物^[2]^。但是蒽环类和紫杉烷类药物在辅助和新辅助治疗中的应用逐渐广泛，使转移性乳腺癌的药物选择受到限制^[3]^。

对于蒽环类和紫杉烷类为基础的治疗方案均耐药的患者，卡培他滨是一个可选择的重要的细胞毒药物，并且是被批准在这种情况下使用的唯一药物。既往曾使用蒽环类和紫杉烷类治疗的患者应用卡培他滨单药治疗的有效率可高达28%^[4-6]^。该药还具有口服给药的优势且很少发生骨髓抑制。蒽环类和紫杉烷类耐药的患者可选择的其他药物包括：吉西他滨、长春瑞滨和聚乙二醇脂质体多柔比星（PLD）等^[7]^。

另外，生物治疗也正逐渐进入到转移性乳腺癌治疗的临床应用之中^[1]^。对于HER-2阳性的肿瘤患者，曲妥珠单抗取得了成功，成为该亚群患者的标准治疗方案的核心药物。仍未发现对HER-2阴性的肿瘤患者特别有效的药物，曾一度成为热点的抗血管生成药物贝伐单抗目前在转移性乳腺癌中的治疗作用受到了许多质疑。

**2. 卡培他滨与转移性乳腺癌的治疗**

一些临床研究评价了卡培他滨联合治疗或作为单药在转移性乳腺癌中的疗效。卡培他滨联合多西他赛被批准应用于既往含蒽环类化学治疗失败的转移性乳腺癌患者^[8]^。此外，卡培他滨作为单药治疗被批准应用于对紫杉醇和含蒽环类化疗均耐药的转移性乳腺癌患者，或对紫杉醇耐药且无进一步应用蒽环类指征的患者。卡培他滨给药的标准是每天2500 mg/m^2^ bid连用二周，然后休息一周。

一些先前的Ⅱ期临床研究显示了卡培他滨单药在既往应用过紫杉类或蒽环类方案治疗的患者中的有效性^[4,5,9,10]^。在这些研究中，卡培他滨单药的总有效率为15-28%，中位无进展生存期是3.1-4.9个月。近来的数个III 期研究也很好地阐述了卡培他滨单药或联合治疗的有效性和安全性。

一项大型的 III 期临床试验在蒽环类和紫杉类药物耐药的752 例患者中比较了卡培他滨单药与卡培他滨联合Ixabepilone 的疗效和不良反应^[11]^。单药卡培他滨的中位疾病至进展时间（PFS）4.2月，有效率为14%。联合治疗组虽然在中位PFS 和有效率较单药组有所提高（5.8月和35%），但是同时也显著增加了毒副作用。3/4度感觉神经毒性、乏力和粒缺在联合治疗组分别达到了21%、9%和68%，而单药组为0%、3%和11%。

另一项著名的III 期临床试验RIBBON-2 在蒽环类方案或紫杉类方案耐药的患者中展开^[12]^，462例患者随机分组到卡培他滨单药或联合贝伐单抗治疗组。结果显示虽然联合组的治疗有效率高于单药组（19.8%比9.1%），然而并未转化为疾病至进展时间（PFS）和总生存（OS）的优势，两组的PFS 和OS 相当（PFS: 4.86月比4.17月； OS: 15.1月比14.5月）。

所有的临床数据均表明，卡培他滨单药治疗具有良好的安全性，且很少发生骨髓抑制或脱发^[13]^。最常见的不良事件是手-足综合征，这并不威胁生命且能通过剂量调整有效控制。卡培他滨也与消化道不良事件的发生有关（如腹泻、口腔炎），但这些不良事件一般为轻中度。卡培他滨的给药剂量减至2000mg/m2以下则3/4级腹泻的发生率较标准的2500mg/m2方案低^[14]^。重要的是，卡培他滨剂量减少并不影响疗效^[5]^。

当然，卡培他滨也被作为一种与包括Herceptin 或贝伐单抗在内的生物制剂的联合用药而加以研究。但在Her-2 阴性患者中与贝伐单抗的联合未取得预期的疗效，因此，该药与其他有效低毒药物的联合方案的探索仍然是一个值得研究的方向。

**3. 万特普安®及其在乳腺癌方面的应用**

**万特普安®**

铜绿假单胞菌注射液（商品名万特普安®）是一种基因工程技术制备的新型菌类制剂，其药物成分是具有自主知识产权的“铜绿假单胞菌-甘露糖敏感血凝菌毛株”（PA-MSHA）。铜绿假单胞菌注射液于1998年正式批准上市，已经在临床使用十余年，广泛用于包括乳腺癌在内的多种恶性肿瘤的辅助治疗。长期的临床应用表明，铜绿假单胞菌注射液不但能显著抑制肿瘤的生长，还能有效防止肿瘤的复发和转移，延长肿瘤患者的生存期并提高生活质量^[15,16]^。

**万特普安®的作用机制**

2009年，复旦大学附属肿瘤医院邵志敏教授的研究团队对万特普安®治疗乳腺癌的作用机理进行了深入研究。体外和体内研究发现，万特普安®对于乳腺癌细胞（MDA-MB-231HM和MDA-MB-468细胞）具有直接的靶向杀伤作用，其特有的MSHA菌毛能够与乳腺癌细胞表面EGFR受体上的甘露糖分子特异性结合，阻断EGFR受体及其胞内信号分子（AKT、ERK）的活化，进而抑制乳腺癌细胞的生长并诱导其凋亡；另一方面，万特普安®还能够显著降低乳腺癌细胞所产生的与肿瘤转移和血管生成相关的蛋白水平（MMP、E-Cadherin、VEGF）；裸鼠乳腺癌移植瘤的生长得到了有效控制，肺转移率和肺转移灶的数量也显著低于对照组。该研究成果已经发表在2009年的《Journal of Cellular Biochemistry》和2010年《Oncogene》杂志上，这为万特普安®用于乳腺癌的临床治疗提供了充分的理论依据^[17,18]^。

另一方面，万特普安®还是一种高效的免疫激活剂。2008年的一项研究发现，万特普安®所具有的MSHA菌毛能够充分活化免疫细胞表面的Toll样受体（TLR4）^[19]^，诱导单核细胞（树突状细胞、巨噬细胞）分化成熟，并进一步激活T淋巴细胞（CTL）和NK细胞，诱导抗肿瘤的特异性和非特异性免疫反应，帮助机体重建肿瘤免疫监视并改善肿瘤细胞所处的微环境^[20]^。因此，万特普安®是一种兼具“靶向治疗”和“免疫治疗”双重作用机制的抗肿瘤新药。万特普安®联合化疗不但能够发挥自身的靶向治疗作用的优势，同时也能改善化疗所造成的机体免疫功能低下，提高肿瘤细胞对化疗的敏感性，并可能减轻化疗的毒副作用。

**万特普安®在乳腺癌治疗中的应用**

万特普安在乳腺癌治疗中研究较多的是在新辅助化疗中的应用。2009年，天冿大学附属肿瘤医院进行的单中心临床研究中，女性局部晚期乳腺癌患者（Ⅱ~Ⅲ期）40例，随机分为两组，试验组采用TE化疗方案联合万特普安®皮下注射，而对照组单用TE化疗方案，化疗4个周期后行根治或保乳手术治疗。研究结果表明，试验组的总有效率（RR）85%，显著高于对照组60%（*P*<0.05）；试验组的病理缓解率（pCR）为30%，也高于对照组的10%。患者的体力状况，试验组治疗后的Karnofsky评分显著高于治疗前，也高于对照组治疗后的评分（*P*<0.01）。患者的免疫学指标，试验组IFN-γ和IL-2的血清浓度经治疗后升高，IL-4和IL-10的血清浓度降低，治疗前后比较均有显著差异（*P*<0.05）；治疗后试验组与对照组上述指标比较差异有统计学意义（*P*<0.01）。肿瘤细胞凋亡与转移相关蛋白水平，试验组治疗后血清Caspase-3浓度升高，VEGF、MMP-2和MMP-9血清浓度降低（*P*<0.05）。安全性评价表明，试验组与对照组均未出现严重的髓外不良反应和急性心脏毒性^[21]^。

2009年，中南大学湘雅二医院进行的单中心临床研究中，60例女性局部晚期乳腺癌患者（T2~T4）60例，随机分为两组，试验组采用TAC化疗方案联合万特普安®皮下注射，每日1次，连用21天；而对照组单用TAC化疗方案，根据治疗效果进行2~4个周期的化疗后手术。研究结果表明，化疗2个周期后，试验组的总有效率（RR）76.7%，显著高于对照组，化疗3、4个周期后仍有显著性差异。患者体力状况，试验组治疗前后的Karnofsky评分无显著性差异，而对照组治疗后Karnofsky评分显著低于治疗前。化疗的毒性方面，试验组和对照组最常见的毒性反应均为恶心呕吐、骨髓抑制以及脱发，但试验组较对照组发生率有明显的减少，而毒性反应程度亦有明显的减轻^[22]^。

以上研究结果表明，在局部晚期乳腺癌患者进行新辅助化疗时，联合万特普安®皮下注射，能够有效提高患者的病理缓解率（pCR）和总有效率（RR），减少化疗周期，同时改善患者的体力状况，并降低由化疗所引起的毒性反应。

**万特普安®用于HER-2受体阴性乳腺癌的治疗**

在天冿大学附属肿瘤医院进行的临床研究中，试验组20例患者经过化疗与万特普安®联合治疗后，有6例在术前达到pCR（30%）。对受试病例进行受体分型分析发现，试验组6例达到pCR的患者全部为HER-2受体阴性，其中一例为三阴性乳腺癌，提示万特普安®对于HER-2受体阴性的局部晚期乳腺癌可能具有更好的疗效；在基础研究方面，复旦大学附属肿瘤医院邵志敏教授的研究中，对万特普安®的靶向杀伤作用相对敏感的MDA-MB-231HM和MDA-MB-468细胞株也不表达HER-2受体。综上，临床研究与基础研究结果均提示了万特普安®对于HER-2受体阴性乳腺癌的治疗价值。

乳腺癌是一种异质性疾病，不同亚型对化疗的敏感性及预后都不尽相同。HER-2受体阴性的乳腺癌，特别是HER-2阴性、ER阳性的Luminal A型乳腺癌患者，新辅助化疗所能达到的pCR率最低；即使对pCR率相对较高的三阴性乳腺癌患者，其远期预后也不理想。例如在复旦大学附属肿瘤医院进行的PCb每周方案新辅助化疗研究中，ER阴性、PR阴性、HER-2阳性或三阴性的乳腺癌患者的pCR率较高，分别为32.6%、30.6%、33.3%和33.3%，而ER阳性或Luminal A型（ER阳性、HER-2阴性）乳腺癌的pCR率较低，仅有9.7%和8.3%，因此，必须根据不同类型乳腺癌的生物学行为给予相应的治疗^[23]^。总体来说，对于HER-2受体阴性的乳腺癌患者，不但新辅助化疗效果较差，而且由于不能使用赫赛汀（Herceptin），也进一步限制了这类患者的临床治疗手段和治疗效果。因此，HER-2受体阴性的乳腺癌是治疗中的难点，这类患者迫切需要探索新的治疗手段。

通过以上的分析，将万特普安®用于HER-2受体阴性乳腺癌治疗可能显著提高HER-2受体阴性乳腺癌患者的有效率，无进展生存期（PFS）和总生存期（OS）。鉴于万特普安在HER-2阴性的乳腺癌新辅助治疗中的卓越表现，本研究拟探索将其联合希罗达治疗蒽环类和/或紫杉类方案耐药的转移性乳腺癌中的治疗地位。

**二、研究方法**

本研究是一项单臂、开放、单中心II期临床研究。主要目的是评价希罗达联合万特普安解救治疗HER-2阴性的转移性乳腺癌的有效性和安全性。

**三、入排标准**

**1. 入选标准**

1) 女性，年龄18~70岁；

1. 组织病理学确诊为浸润性乳腺癌，并发生转移不适合行局部治疗的患者；
2. 病理组织学检查确定HER-2表达呈阴性的患者（免疫组化检测HER-2为-或+，若HER-2为++，则需进行FISH检测，结果为阴性）；
3. 经蒽环类和/或紫杉类方案治疗后进展，适合行希罗达治疗的患者；
4. 既往未用过含希罗达的方案治疗或含希罗达方案治疗有效，停化疗4月以上疾病发生进展的患者；
5. Karnofsky评分≥70；ECOG评分0~2；
6. 心脏、肝脏、肾脏及骨髓造血功能良好；
7. 实验室检查符合WBC≥4×10^9^/L；Hb≥90 g/L；plt≥100×10^9^/L；
8. 患者自愿参加，签署知情同意书。

**2. 排除标准**

1. 妊娠或哺乳期妇女；
2. 同时还患有其他肿瘤者；
3. 最近1个月内接受其他类似药物临床试验；
4. 有严重未控制的内科疾病或急性感染者；
5. 已知对本研究药物或既往多种药物过敏者；
6. 患者不能理解研究的目的或不同意研究的要求；
7. 其他研究者认为不适合入本研究组的患者。

**四、治疗方案**

经组织病理学确诊为HER-2受体阴性转移性乳腺癌女性患者96例入组本研究。用药方案：希罗达1000 mg/m^2^ Bid，PO，d1-14，21天为一个周期。口服希罗达同时，从希罗达开始第一天起皮下注射万特普安（建议腹部和健侧上臂皮下注射），首次注射剂量为0.5 ml，以后1 ml/次，隔天一次连续给药，直至疾病进展或发生不可耐受的毒性。

**五、疗效和和不良反应评估**

**1. 疗效评估**

疗效评估参照实体瘤疗效评定标准（RECIST 1.1版），每两个疗程后进行疗效评估。（详见附录三）

**2. 安全性评估**

在每个化疗周期结束后，都应根据临床和实验室检查结果，参照NCI CTC3.0常见毒性反应标准，对可能存在的不良反应进行评估。

**六、检查方法**

**1. 入组前检查**

所有病例在入组前应完成下列各项检查内容：

1. 病史采集：包括年龄、性别、既往病史、过敏史、入院日期、入院主诉等；
2. 全身体格检查：包括身高、体重、体表面积和生命体征检查（体温、血压、心率、呼吸、脉搏）；
3. 血常规（RBC、WBC、PLT、Hb）
4. 尿常规（PRO、WBC、RBC）
5. 心电图检查
6. 肝功能（TP、TBIL、ALT、AST、AKP、A/G、LDH）
7. 肾功能（BUN、Cr）
8. 影像学检查：胸部CT、腹部MRI、全身骨扫描等。
9. 免疫学检查（CD3、CD4/CD8、NK、IL-2、IFN-γ、IL-4、IL-10、肿瘤浸润免疫细胞）
10. 肿瘤转移相关指标检查（MMP-9、VEGF、E-Cadherin）
11. 体力状况评分：参照KPS或ECOG评分标准。

**2. 治疗阶段的检查**

每个疗程化疗开始之前都要进行实验室检查，包括血常规、肝功能、肾功能，同时进行常规体检和体力状况评分。

治疗过程中每周监测血常规，必要时调整化疗药的剂量；如出现粒细胞减少时可应用粒细胞集落刺激因子（G-CSF）进行支持治疗。

第2个疗程化疗后进行胸部CT、腹部MRI、肿瘤相关标记指标（CEA、CA15-3、CA125）检查，并根据RECIST 1.1标准评价疗效。

在研究过程中取得的血样标本还将用于进行相关的肿瘤转移相关指标（MMP-9、VEGF、E-Cadherin）、免疫学指标（CD3、CD4、CD8、NK）和肿瘤浸润免疫细胞（TAM、Treg）的检测。

**七、合并用药和伴随治疗**

**1. 内分泌治疗**

原则上不因同时应用内分泌治疗药物，但允许应用内分泌药物作为支持治疗，如应用孕酮类药物的改善肿瘤患者恶液质的治疗。

**2. 双膦酸盐**

在检测肾功能正常时双膦酸盐的使用是允许的，在研究过程中开始双膦酸盐治疗但无疾病进展其他证据的患者不被认为是疾病进展。

**3. 姑息性放疗**

允许在治疗中应用姑息性放疗作止痛等处理，但因疾病进展而导致的放疗应作为疾病进展而出组。

**4. 对症支持治疗**

所有患者均可接受对症支持治疗，包括输血及血小板、升白细胞、使用抗生素及止吐治疗。

1）集落刺激因子

集落刺激因子G-CSF可以用来治疗化疗相关性中性粒细胞减少，但不推荐用于预防性治疗。III及IV度中性粒细胞减少时应停用化疗药物希罗达而给予G-CSF治疗。出现发热性中性粒细胞减少应遵循相应的治疗指南。

2）输注血小板和/或升血小板治疗

当出现Ⅱ度或以上血小板下降时，可停用希罗达，而给予输注血小板和/或白介素－11（IL－11）、TPO等治疗。

3）伴随用药

在本研究中应尽量减少伴随用药量。但若涉及到患者的利益且不干扰本研究时，研究者可根据具体情况使用。在研究进行中（包括初次给药前4周内）的所有伴随用药均应在病历及病例报告表中记录。

**八、终点指标**

**1. 主要终点：**无疾病进展生存期（PFS）

**2. 次要终点：**疾病缓解率（ORR）、总生存期（OS）、安全性、肿瘤转移相关指标、免疫学指标。

**九、药品使用方法及注意事项**

**希罗达**

为了降低毒性至基线时的水平或0~1级，允许给药延迟至3周（如果有潜在或可预测的获益，则也可允许延迟更长时间）。但是，应仅在出现严重的毒性反应时减少卡培他滨的剂量（暂时的或永久的），包括：≥2级的毒性反应如腹泻、尽管采取了预防措施仍然出现的持续性恶心/呕吐、手-足综合征（HFS）、高胆红素血症（＞3×ULN）或肝酶升高（AST或ALT＞2.5 ×ULN）。

一旦患者进行了卡培他滨的减量，则在以后周期的卡培他滨剂量不再增加。卡培他滨剂量中断造成治疗缺失时，之后的治疗应继续按计划时间表进行。由于治疗中断造成的卡培他滨剂量的遗漏不应再补足。此外，如果休息期的延长超过了下一次规定的治疗周期时，重新开始卡培他滨治疗时应给予14天的完整剂量，且研究治疗的时间表应按原计划进行。

根据体表面积计算标准剂量和由于休息期延长而中断的卡培他滨的剂量减少请参考附录五。

**供药与储存**

卡培他滨以500mg薄膜包衣片的形式供药并根据研究方案的给药说明通过口服给药。该片剂没有划痕，不能掰裂。

储存温度不要超过30℃。

卡培他滨将根据法律要求和GMP规范贴上标签。

给药说明

- 卡培他滨将以1000 mg/m^2^ 每天两次，在每3周为一个周期的第1~14天给药
- 在给药期间，应指导患者在饭后（早餐和晚餐）30分钟内以水吞服药片
- 卡培他滨每日剂量是根据体表面积（BSA）决定的，请应用附录五确定适合的剂量及所需药片的数量。

**万特普安®**（铜绿假单胞菌注射液）

- 厂家：北京万特尔生物制药有限公司；
- 性状：乳白色液体，有微粒，无杂物；
- 规格：一次性预充注射器，1 ml/支；
- 贮藏：2~8 ^o^C避光保存。
- 用法：皮下注射，首次注射剂量为0.5 ml，以后1 ml/次，隔天注射一次（qod）。

使用注意事项：

1. 有过敏史者慎用。尤其已知既往多种药物过敏史者，建议不入组。
2. 存放后有少量沉淀，使用时需将冷藏药液恢复至室温并充分摇匀，不应有摇不散的凝块或异物。
3. 不得与其他药液混合注射。药液一定要注入皮下，注入皮内可致严重的局部反应，注射速度尽量缓慢。
4. 一次性预充注射器包装，不得分次使用。
5. 皮下注射后少数患者可能会出现局部红肿、疼痛等现象，一般不需要处理，可自行消退。注射第二针时应换另侧部位。如出现皮下硬结或症状严重者建议局部热敷，以促进药液吸收。也可脐周、健侧上臂皮下轮流注射。
6. 注意休息多饮水、注意保暖可减少不良反应发生。如发热高于38.5℃，排除其它原因后可进行对症处理，但最好用物理降温法。用解热镇痛药（如消炎痛栓剂或贴膏剂）对症处理能很快缓解。
7. 若有不可耐受的毒性，可减量为每周2次用药。
8. 如有患者出现副反应（如高热、寒战）建议加强临床护理，具体方法由医生根据临床经验处理，建议可预先使用解热镇痛药预防。

**十、试验的中止**

试验中止是指临床试验按方案尚未结束，中途因为以下原因停止全部试验。主要目的是保护受试者权益，保证试验质量，避免不必要的经济损失。

- 出现疾病进展；
- 出现无法接受的不良事件；
- 因毒性调整剂量2次及以上仍不能耐受者；
- 研究者认为受试者有必要停止试验；
- 受试者主动要求退出试验；
- 受试者治疗延迟时间超过3周；
- 受试者在未完成全部治疗前不再接受用药或检查而失访；
- 受试者死亡。

**十一、不良事件的处理及报告**

**1. 不良事件**

不良事件（AE）是指病人或临床试验受试者接受一种药品后出现的不良医学事件，但并不一定与治疗有因果关系。

**2. 不良事件报告期**

为随机化开始至最后一次访视之间，在此期间发生的不良事件，无论与研究药物是否有关，都应填入病例报告表。另外，任何在不良事件报告期后发生的不利事件，研究者估计可能与研究药物有关，也应作为不良事件报告。

**3. 严重不良事件（SAE）**

不良事件符合下列一项或几项标准时定义为严重不良事件：

- 死亡；
- 有生命危险；
- 导致住院治疗或住院时间延长；
- 永久或严重致残；
- 先天畸形/缺陷、重要的医学事件。

**4. 严重不良事件的报告途径**

对于所有的严重不良事件，必须立即停止试验，采取保护受试者的相应措施。研究者必须在24小时内通过电话或传真将严重不良事件报告申办者。研究者应对严重不良事件追踪至解决。有关医学文件均应记录在原始文件中，包括实验室检查结果报告单（如：X线检查、心电图等）。

**5. 记录与报告**

研究者应向患者详细说明，要求患者如实地反映用药后的病情变化。医师要避免诱导性提问。在观察疗效的同时，密切注意观察不良事件，分析原因，作出判断，并追踪观察和记录，统计不良反应发生率。

对试验期间出现的不良事件，应将其出现的时间、症状、程度、持续时间、处理措施、转归等记录于病例报告表，评价其与试验药物的相关性，并由研究者详细记录，签名并注明日期。不良事件的严重度将根据NCI CTC3.0版分级。对每个症状，应报告自上次随访以来发生不良事件的最高分级。

不良事件与试验药物关系的判定：按5级判定：即肯定有关、可能有关、可能无关、无关、无法评定。前两者计为不良反应，统计不良反应的发生率。

- 肯定有关：反应出现符合用药后合理的时间顺序，反应符合所疑药物已知的反应类型；停药后改善，重复给药再出现该反应。
- 可能有关：反应出现符合用药后合理的时间顺序，反应符合所疑药物已知的反应类型；病人的临床状态或其它治疗方式也可能产生该反应。
- 可能无关：反应出现不太符合用药后合理的时间顺序，反应不太符合所疑药物已知的反应类型；病人的临床状态或其它治疗方式有可能产生该反应。
- 无关：反应出现不符合用药后合理的时间顺序，反应有符合非试验药物已知的反应类型；病人的临床状态或其它治疗方式可能产生该反应，疾病状态改善或停止其它治疗方式反应消除，重复使用其它治疗方法反应出现。
- 无法评定：反应出现与用药后的时间无明确关系，与该药品已知的反应类型相似，同时使用的其它药物也可能引起相同的反应。

不良事件与试验药物的关系

|  | 肯定有关 | 很可能有关 | 可能有关 | 可疑 | 不可能有关 |
| --- | --- | --- | --- | --- | --- |
| 与试验用药有合理的时间顺序 | + | + | + | + | — |
| 已知的药物反应类型 | + | + | + | — | — |
| 停药后反应减轻或消失 | + | + | ± | ± | — |
| 再次给药后反应反复出现 | + | ？ | ？ | ？ | — |
| 无法用受试者疾病来解释 | + | + | — | ± | — |

**十二、样本量**

希望加入万特普安后，PFS 由希罗达单药的4.2个月提高到联合治疗的5.7个月，a=0.05, power 85%, 需要样本量为88例，加上10%的脱落，需入组96例。

**十三、数据处理**

**1. 研究者填写数据要求**

1. 对所有填写了知情同意书并筛选合格进入试验的患者，均须认真而详细地记录病例报告表中的任何项目，不得空项或漏项（无记录的空格划横线）；
2. 病例报告表中所有数据需与受试者病历数据核对，保证无误；
3. 病例报告表作为原始数据，做任何更正时只能划线，旁注改后数据，并有研究者签名标注日期；
4. 化验单复印件粘贴在病例报告表后的化验单粘贴处；
5. 对显著偏高或在临床接受范围以外的数据，须加以核实，由研究者做必要说明；
6. 请参照病例报告表填写说明。

**2. 监查员监察数据要求**

1. 监查员在试验过程中要定期到各试验中心检查受试者的知情同意及筛选纳入情况；
2. 确认所有病例报告表填写正确并与原始资料一致；
3. 所有错误或遗漏均已改正或注明，经研究者签名并注明日期；
4. 每一受试者的剂量改变、治疗变更、合并用药、间发疾病等均应确认并记录；
5. 核实入选受试者的退出与失访均在病例报告表中予以说明；
6. 确认所有不良事件均已记录在案，严重不良事件已作出报告记录在案；
7. 核实试验用药品是否按照有关规定进行供应、储藏、分发、收回，并做相应的记录；
8. 每个入选病例都必须完成CRF，完成的原始CRF为北京万特尔生物制药有限公司所拥有，未经本公司书面同意，不得以任何形式提供给第三者，除非国家药品监督管理局要求；
9. CRF表由研究者填写，每个入选病例必须完成病例报告表。完成的CRF表由临床监查员审查后，第一联移交数据统计单位，进行数据录入与管理工作，第二联交申办单位存档，第三联由各中心临床药理基地存档。

**3. 数据的可溯源性、病例报告表（CRF）的填写与移交**

最原始记录为研究病历以便妥善保存。病例报告表来自研究病历，由研究者填写，每个入选病例必须完成病例报告表。完成的病例报告表由临床监查员审查后，第一联交数据统计单位，进行数据录入与管理工作。第一联移交后，病例报告表的内容不再作修改。

**4. 数据的录入与修改**

数据录入与管理由统计单位数据管理员负责。采用Epidata软件建立专用数据库，进行数据录入与管理。为保证数据的准确性，应由两个数据管理员独立进行双份录入并校对。

对病例报告表中存在的疑问。数据管理员将填写疑问解答表（DRQ），并通过临床监查员向研究者发出询问，研究者应尽快解答并返回，数据管理员根据研究者的回答进行数据修改，确认与录入，必要时可以再次发出DRQ。

**5. 数据的锁定**

在数据审核并确认建立的数据库正确后，由主要研究者、申办者、统计分析人员和药品监督管理人员对数据进行锁定。锁定后的数据文件不可再做改动。数据锁定之后发现的问题，经确认后可在统计分析程序中进行修正。

**十四、统计分析**

由专业统计学工作者承担统计分析任务，并参与从试验设计、实施至分析总结的全过程。试验方案和病例报告表完成后制定统计分析计划，并在试验过程中根据需要进行必要的修改，数据锁定前完成统计分析计划，数据分析完成后提供统计分析报告。

**1. 分析数据集**

1. **全分析集**（FUll Analysis Set-FAS）：指合格病例和脱落病例的集合，但不包括剔除病例。
2. **符合方案数据集**（Per-Protocol population，PP）：指符合纳入标准并完成治疗方案的病例集合，即符合试验方案、依从性好、未服用禁用药物、完成CRF规定填写内容的病例。
3. **安全数据集**：至少接受一次治疗，且有安全性指标记录的实际数据，退出病例不作数据接转。

**2. 统计方法**

**统计描述**

1. 是否符合正态分布：不符合时修改统计方法或进行数据转换；
2. 有无离群值：进行统计和专业的分析，决定取舍；
3. 有无缺失值：当个别受试者某一主要疗效指标未能测到时，进行末次观察数据接转。
4. 脱落病例所占的比例：不宜大于20%，否则应加以分析说明；
5. 描述性统计分析：指出均数，标准差，最大值，最小值，可信区间，率等。

**3. 分析方法**

1. 计量资料：采用t检验、配对t检验、秩和检验、配对秩和检验等方法；
2. 计数资料：采用Fisher确切概率法等；等级资料采用秩和检验。
3. 疗效指标的分析：计数资料采用CMH卡方检验或logistic回归；计量资料根据资料特点采用方差分析或秩和检验；生存资料采用Kaplan-meier方法或Cox回归。
4. FAS分析和PP分析：对主要疗效指标同时进行PP分析和FAS分析。

**4. 统计表达**

1. 报告主要采用表格表示，表格具有自明性，即具有表题，表注，例数。
2. 一般采用双侧检验，P小于或等于0.05者将被认为所检验的判别有统计意义。

**5. 统计软件**

采用SAS软件分析。

**6. 检验类型**

优效性检验。

**7. 统计分析内容**

统计分析计划书由统计人员撰写，包括数据管理、统计方法和分析内容。由统计结果书写统计报告书，包括各试验单位及汇总数据的统计报告书，以多种表格组成。并在正式统计分析前加以确认和细化。主要分析内容包括：

1. 两组病例分布：两组总脱落率和由于不良事件而脱落的脱落率的比较，将采用卡方检验。
2. 可比性分析：比较人口学资料和其他基础值指标，以衡量两组的可比性。
3. 依从性分析：比较两组病人是否按时按量使用试验药物，未用方案中禁用的药物和食物。
4. 有效性分析：主要指标和全局性指标采用PP和FAS分析；由于本研究是多中心临床试验，分析时应考虑中心效应对疗效指标的影响。
5. 安全性分析：首先根据不良反应相关性的要求，列表描述两组的不良事件和不良反应（包括各种不良事件的例数、实验室检测指标在试验前后“正常转异常”的例数和转异率）。采用卡方检验对不良反应进行统计分析。

**十五、质量控制和保证**

在试验过程中，将定期对试验中心进行监查访问，以保证试验方案执行。复核原始资料以确认病例报告表上的数据的一致性。

**十六、伦理规范**

本研究过程必须严格按照SFDA《药物临床试验质量管理规范》和《赫尔辛基宣言》的要求进行。

**伦理委员会（IEC）**

临床研究开始前，试验方案、知情同意书以及提供给受试者的其他资料都须经过组长单位伦理委员会的审核批准，并将相关批件提供给审办者。

**知情同意书（ICF）**

研究者有责任在每位受试者入选研究前，给予口头和书面上全面告知此研究的目的、过程以及潜在风险等信息，告知受试者有权决定是否参加和在任何时候都可以退出试验。受试者或其法定代理人阅读并理解后签署知情同意书，并保留签字页复印件。

**十七、试验进度、资料保存和总结报告**

**1. 试验进度**

2011年3月~2013年3月

**2. 资料保存**

病例报告表应由主要研究者签字复核，试验完成后，所有病例报告表、典型病例的详细资料和临床试验药品使用记录表应该保存。研究者应保存所有受试者相关原始资料、保存获CR或PR患者的相关影像学资料、已签署的知情同意书原件、CRF复印件和药物发放记录。

**3. 总结报告**

资料的统计结果交由研究单位确认并作为撰写“临床试验小结报告”的依据，完成后的小结报告盖章研究单位1份存档。

**4. 试验结果的发表**

关于本次研究的有关数据必须由研究单位发表。

**参考文献**

1. National Comprehensive Cancer Network. Clinical Practice Guidelines in　Oncology: Breast cancer v.2.2008. http://www.nccn.org/professionals/physician_gls/PDF/breast.pdf. (2008).
2. Bernard-Marty,C., Cardoso,F. & Piccart,M.J. Facts and controversies in systemic　treatment of metastatic breast cancer. Oncologist. 9, 617-632 (2004).
3. Verma,S. & Clemons,M. First-line treatment options for patients with HER-2　negative metastatic breast cancer: the impact of modern adjuvant chemotherapy.　Oncologist. 12, 785-797 (2007).
4. Blum,J.L. et al. Multicenter phase II study of capecitabine in paclitaxel-refractory　metastatic breast cancer. J. Clin. Oncol. 17, 485-493 (1999).
5. Blum,J.L. et al. Multicenter, Phase II study of capecitabine in taxane-pretreated　metastatic breast carcinoma patients. Cancer 92, 1759-1768 (2001).
6. Fumoleau,P. et al. Multicentre, phase II study evaluating capecitabine　onotherapy in patients with anthracycline- and taxane-pretreated metastatic　breast cancer. Eur. J. Cancer 40, 536-542 (2004).
7. O'Shaughnessy,J., Twelves,C. & Aapro,M. Treatment for anthracyclinepretreated　metastatic breast cancer. Oncologist. 7 Suppl 6, 4-12 (2002).
8. O'Shaughnessy,J., et al. Superior Survival With Capecitabine Plus Docetaxel Combination Therapy in Anthracycline-Pretreated Patients With Advanced Breast Cancer: Phase III Trial Results. J Clin Oncol. 20, 2812-2823 (2002).
9. Fumoleau,P. et al. A multicentre phase II study of the efficacy and safety of　docetaxel as first-line treatment of advanced breast cancer: report of the Clinical　Screening Group of the EORTC. Ann. Oncol. 7, 165-171 (1996).
10. Reichardt,P. et al. Multicenter phase II study of oral capecitabine (Xeloda(")) in　patients with metastatic breast cancer relapsing after treatment with a taxanecontaining　therapy. Ann Oncol 14, 1227-1233 (2003).
11. Thomas, ES., et al. Ixabepilone Plus Capecitabine for Metastatic Breast Cancer Progressing After Anthracycline and Taxane Treatment. J Clin Oncol, 25, 1-7 (2007).
12. Miller, KD. et al. Randomized Phase III Trial of Capecitabine Compared With Bevacizumab Plus Capecitabine in Patients With Previously Treated Metastatic Breast Cancer. J Clin Oncol, 23,792-799 (2005).
13. Gelmon,K., Chan,A. & Harbeck,N. The role of capecitabine in first-line treatment　for patients with metastatic breast cancer. Oncologist. 11 Suppl 1, 42-51 (2006).
14. Bajetta,E. et al. Safety and efficacy of two different doses of capecitabine in the　treatment of advanced breast cancer in older women. J. Clin. Oncol. 23, 2155-2161 (2005).
15. 牟希亚等. 绿脓杆菌甘露糖敏感血凝菌毛株. 发明专利，专利号ZL200510059850.X
16. 牟希亚，郭雁群，牟心赤. 菌毛学研究进展. 大连出版社1999.
17. Zhebin Liu, Yifeng Hou, Min Dong, et al. PA-MSHA inhibits proliferation and induces apoptosis through the up-regulation and activation of caspases in the human breast cancer cell lines [J]. J Cell Biochem 2009, 9999:1-12.
18. Zhebin Liu, Yifeng Hou, Jie Zhu, et al. PA-MSHA Inhibits Her-1/EGFR Signaling, Invasiveness and the Metastasis Potential of Breast Cancer Cells both in Vitro and in Vivo Mediated by Type I Pili via a Mannose-Dependent Way [J]. Oncogene. Revision.
19. da Silva Correia J, Ulevitch RJ. MD-2 and TLR4 N-linked glycosylations are important for a functional lipopolysaccharide receptor [J]. J Biol Chem. 2002 Jan 18;277(3):1845-54.
20. 孙文平，付红文，刘妮，吴毓，牟希亚，靳岩，高小平.PA-MSHA菌毛株疫苗对三种癌症患者免疫疗效的观察[J].中华微生物学和免疫学杂志. 2000，20(4): 373-376.
21. 毛启新等.铜绿假单胞菌注射液联合新辅助化疗治疗乳腺癌的临床研究[J]. 中华肿瘤防治杂志. 2010
22. 徐峰，唐中华，李允山等.铜绿假单胞菌制剂在乳腺癌新辅助化疗中的应用[J].中南药学. 2009，7(8): 626-629.
23. 陈小松.紫杉醇联合卡铂每周方案在乳腺癌新辅助化疗中的疗效及安全性研究. 复旦大学硕士学位论文. 2009.

**附录一：KPS和ECOG评分标准**

| **Karnofsky（KPS）** |  |  | **Zubrod-ECOG-WHO** |
| --- | --- | --- | --- |
| 正常，无症状及体征 | 100 | 0 | 正常活动 |
| 能进行正常活动，有轻微症状及体征 | 90 | 1 | 有症状，但几乎完全可自由活动 |
| 勉强可进行正常活动，有一些症状或体征 | 80 |  |  |
| 生活可自理，但不能维持正常生活或工作 | 70 | 2 | 有时卧床，但白天卧床时间不超过50% |
| 有时需人扶助，但大多数时间可自理 | 60 |  |  |
| 常需人照应 | 50 | 3 | 需要卧床，卧床时间白天超过50% |
| 生活不能自理，需特别照顾 | 40 |  |  |
| 生活严重不能自理 | 30 | 4 | 卧床不起 |
| 病重，需住院积极支持治疗 | 20 |  |  |
| 病危，临近死亡 | 10 |  |  |
| 死亡 | 0 | 5 | 死亡 |

**附录二：NCI-CTC常见毒性分级标准（3.0版）**

| 过敏/免疫 | | | | | | |
| --- | --- | --- | --- | --- | --- | --- |
|  | | 分级 | | | | |
| 不良事件 | 简称 | 1 | 2 | 3 | 4 | 5 |
| 过敏反应/超敏反应（包括药物热） | 过敏反应 | 一过性面部发红或药疹；药物热<38℃（<100.4°F） | 皮疹；面部发红；药疹；呼吸困难：药物热≥38℃（≥100.4°F） | 有症状的支气管痉挛，伴或不伴药疹；需要静脉给药治疗；过敏引起的水肿/血管性水肿；低血压 | 过敏性休克 | 死亡 |
| 备注：具有过敏或超敏反应表现的药疹作为过敏反应/超敏反应（包括药物热）进行分级。  还要考虑：细胞因子释放综合症/急性输液反应 | | | | | | |
| 血液/骨髓 | | | | | | |
|  | | 分级 | | | | |
| 不良事件 | 简称 | 1 | 2 | 3 | 4 | 5 |
| 血红蛋白 | 血红蛋白 | <LLN-10.0g/dL  <LLN-6.2mmol/L  <LLN-100g/L | <10.8-8.0 g/dL  <6.2-4.9 mmol/L  <100-80 g/L | <8.0-6.5 g/dL  <4.9-4.0 mmol/L  <80-65 g/L | <6.5 g/dL  <4.0 mmol/L  <65 g/L | 死亡 |
| 白细胞（所有白细胞） | 白细胞 | <LLN-3000/mm^3^  <LLN-3.0×10^9^/L | <3000-2000/mm^3^  <3.0-2.0×10^9^/L | <2000-1000/mm^3^  <2.0-1.0×10^9^/L | <1000/mm^3^  <1.0×10^9^/L | 死亡 |
| 淋巴细胞减少 | 白细胞减少 | <LLN-800/mm^3^  <LLN-0.8×10^9^/L | <800-500/mm^3^  <0.8-0.5×10^9^/L | <500-200/mm^3^  <0.5-0.2×10^9^/L | <200/mm^3^  <0.2×10^9^/L | 死亡 |
| 中性粒细胞/粒细胞（ANC/AGC） | 中性粒细胞 | <LLN-1500/mm^3^  <LLN-1.5×10^9^/L | <1500-1000/mm^3^  <1.5-1.0×10^9^/L | <1000-500/mm^3^  <1.0-0.5×10^9^/L | <500/mm^3^  <0.5×10^9^/L | 死亡 |
| 血小板 | 血小板 | <LLN-75，000/mm^3^  <LLN-75.0×10^9^/L | <75，000-50，000/mm^3^  <75.0-50.0×10^9^/L | <50，000-25，000/mm^3^  <50.0-25.0×10^9^/L | <25，000/mm^3^  <25.0×10^9^/L | 死亡 |
| 心律失常 | | | | | | |
|  | | 分级 | | | | |
| 不良事件 | 简称 | 1 | 2 | 3 | 4 | 5 |
| 室上性和结性心律失常  －选择：  －房颤  －房扑  －房性心动过速/阵发性房性心动过速  －结性/交界性  －窦性心律失常  －窦性心动过缓  －窦性心动过速  －室上性心律失常NOS | 室上性心律失常  －室上性早博（心房提早收缩；房室结/房室交界区提早收缩）  －室上性心动过速 | 无症状，无需治疗 | 非紧急医学治疗 | 有症状，药物不能完全控制，或需要使用装置控制（如起博器） | 危及生命（如与CHF有关的心律失常，低血压，晕厥，休克） | 死亡 |
| 心脏不良事件总论 | | | | | | |
|  | | 分级 | | | |  |
| 不良事件 | 简称 | 1 | 2 | 3 | 4 | 5 |
| 左室收缩功能异常 | 左室收缩功能异常 | 无症状，休息时射血分数（EF）<60-50%收缩分数（SF）<30-24% | 无症状，休息时EF<50-40%; SF<24-15% | 有症状性慢性心衰，对治疗有反应，EF<40-20%; SF<15% | 难治性慢性心衰或控制效果差，EF<20％；需要左室辅助装置，左室部分切除术或心脏移植之类的治疗 | 死亡 |
| 全身性症状 | | | | | | |
|  | | 分级 | | | | |
| 不良事件 | 简称 | 1 | 2 | 3 | 4 | 5 |
| 疲倦（无力，嗜睡，倦怠） | 疲倦 | 与基线相比，轻度疲倦 | 中度，或导致从事某些ADL有困难 | 重度疲倦，影响ADL | 致残 | － |
| 发热（无中性粒细胞减少，中性粒细胞减少定义是ANC<1.0×10^9^/L | 发热 | 38.0-39.0℃  （100.4-102.2°F） | >39.0-40.0℃  （102.3－104.0°F） | >40.0℃(>104.0°F),时间≤24小时 | >40.0℃(>104.0°F),时间>24小时 |  |
| 体重下降 | 体重下降 | 与基线相比，下降程度为5-<10%;无需治疗 | 与基线相比，下降程度为10-<20%;需要营养支持 | 与基线相比，下降程度≥20％，管饲饮食，或需TPN | － | － |
| 皮肤病/皮肤 | | | | | | |
|  | | 分级 | | | | |
| 不良事件 | 简称 | 1 | 2 | 3 | 4 | 5 |
| 脱发/秃发（头皮或身体） | 脱发 | 稀疏或斑秃 | 彻底 | － | － | － |
| 注射部位反应/外渗改变  还要考虑：过敏反应/超敏反应（包括药物热）；溃疡 | 注射部位反应 | 疼痛；骚痒；红斑 | 疼痛或肿胀，伴炎症或静脉炎 | 严重溃疡或坏死；需要手术治疗 | － | － |
| 骚痒/痒  还要考虑：皮疹/脱皮 | 骚痒 | 轻度或局部 | 剧烈或广泛 | 剧烈或广泛，影响ADL | － | － |
| 皮疹/脱皮  备注：皮疹/脱皮可用于GVHD | 皮疹 | 斑疹或丘疹或红斑，无相关症状 | 斑疹或丘疹或红斑，伴骚痒或其他相关症状；局部脱皮或其他病变，范围<50%体表面积（BSA） | 严重全身红皮病或斑疹，丘疹或水疱；脱皮范围≥50%BSA | 全身性剥落性、溃疡性或大疱性皮炎 | 死亡 |
| 胃肠道 | | | | | | |
|  | | 分级 | | | | |
| 不良事件 | 简称 | 1 | 2 | 3 | 4 | 5 |
| 食欲不振  还要考虑：体重下降 | 食欲不振 | 食欲丧失，饮食习惯没有改变 | 经口腔摄入量改变，但无明显体重下降或营养不良状况；需要经口服补充营养 | 引起体重明显下降或营养不良（如经口腔摄入的卡路里和/或体液不足）；需要静脉输液，管饲饮食或TPN | 危及生命的后果 | 死亡 |
| 结肠炎  还要考虑：出血，GI－选择 | 结肠炎 | 无症状，只有病理或放射影像学改变 | 腹痛，大便带血或粘液 | 腹痛，发热，排便习惯改变伴肠梗阻；腹膜体征 | 危及生命的后果（如穿孔，出血，缺血，坏死，中毒性巨结肠） | 死亡 |
| 腹泻  备注：腹泻包括小肠或结肠来源的腹泻，以及/或造瘘口腹泻  还要考虑：脱水；低血压 | 腹泻 | 与基线相比，每天排便次数增加，增加次数<4次；与基线相比，造瘘口排出量轻度增加 | 与基线相比，每天排便次数增加4-6次；静脉输液<24小时；与基线相比，造瘘口排出量中度增加；不影响ADL | 与基线相比，每天排便次数增加，增加次数≥7次；大便失禁；静脉输液≥24小时；住院；与基线相比，造瘘口排出量重度增加；影响ADL | 危及生命的后果（如血液动力学障碍） | 死亡 |
| 粘膜炎/口炎（临床检查）  －选择：  －肛门  －食道  －大肠  －喉  －口腔 | 粘膜炎（临床检查）  －选择  －咽  －直肠  －小肠  －胃  －气管 | 粘膜红斑 | 片状溃疡或假膜形成 | 融合状溃疡或假膜形成；轻微外伤后出血 | 组织坏死；大量自发性出血；危及生命的后果 | 死亡 |
| 恶心  还要考虑：食欲不振；呕吐 | 恶心 | 食欲丧失，饮食习惯没有改变 | 口腔摄入量减少，没有明显的体重下降、脱水或营养不良；需要静脉输液<24小时 | 口腔摄入的卡路里或液体不足；需要静脉输液、管饲饮食或TPN≥24小时 | 危及生命的后果 | 死亡 |
| 呕吐  还要考虑：脱水 | 呕吐 | 24小时内发作1次 | 24小时内发作2到5次；需要静脉输液<24小时 | 24小时内发作≥6次；需要静脉输液或TNP≥24小时 | 危及生命的后果 | 死亡 |
|  | | | | | | |
| 肝胆/胰腺 | | | | | | |
| 肝功能异常/衰竭（临床） | 肝功能异常 | － | 黄疸 | 胆震颤 | 肝性脑病或昏迷 | 死亡 |
| 备注：黄疸不是一种AE，但肝功能异常或胆管阻塞时，会出现黄疸，它作为肝功能异常/衰竭或胆红素升高的结果进行分级。还要考虑：胆红素（高胆红素血症） | | | | | | |
|  | | | | | | |
| 感染 | | | | | | |
| 发热性中性粒细胞减少（原因不明的发热，没有临床或微生物学记录的感染）（ANC<1.0×10^9^/L,发热≥38.5℃） | 发热性中性粒细胞减少 | － | － | 存在 | 危及生命的后果（如感染性休克，低血压，酸中毒，坏死） | 死亡 |
| 还要考虑：中性粒细胞/粒细胞（ANC/AGC） | | | | | | |
| 感染（有临床或微生物学记录）伴3或4级中性粒细胞（ANC<1.0×10^9^/L） | 感染（有临床记录） | － | 局限性，需要局部治疗 | 需要静脉使用抗生素、抗真菌或抗病毒治疗；需要介入放射或手术治疗 | 危及生命的后果（如感染性休克，低血压，酸中毒，坏死） | 死亡 |
| 备注：“没有感染记录时的发热伴有3或4级中性粒细胞作为发热中性粒细胞减少（原因不明的发热，没有临床或微生物学记录的感染）进行分级  还要考虑：中性粒细胞/粒细胞（ANC/AGC） | | | | | | |
| 代谢/实验室 | | | | | | |
|  | | 分级 | | | | |
| 不良事件 | 简称 | 1 | 2 | 3 | 4 | 5 |
| 酸中毒（代谢性或呼吸性） | 酸中毒 | pH<正常值，但≥7.3 | － | pH<7.3 | pH<7.3伴危及生命的后果 | 死亡 |
| 白蛋白，血清水平低（低白蛋白血症） | 低白蛋白血症 | <LLN-3g/dL  <LLN-30g/L | <3-2g/dL  <30-20g/L | <2g/dL  <20g/L | - | 死亡 |
| ALT,SGPT(血清谷丙转氨酶) | ALT | >ULN-2.5×ULN | >2.5-5.0×ULN | >5.0-20.0×ULN | >20.0×ULN | － |
| AST，SGOT（血清谷草转氨酶） | AST | >ULN-2.5×ULN | >2.5-5.0×ULN | >5.0-20.0×ULN | >20.0×ULN | － |
| 胆红素（高胆红素血症） |  | >ULN-1.5×ULN | >1.5-3.0×ULN | >3.0-10.0×ULN | >10.0×ULN | － |
| 肌酐 | 肌酐 | >ULN-1.5×ULN | >1.5-3.0×ULN | >3.0-6.0×ULN | >6.0×ULN |  |
| 备注：对于儿科患者，根据相应年龄的水平进行调整 还要考虑：肾小球滤过率 | | | | | | |
| GGT（γ-谷氨酰转肽酶） | GGT | >ULN-2.5×ULN | >2.5-5.0×ULN | >5.0-20.0×ULN | >20.0×ULN | － |
| 蛋白尿 | 蛋白尿 | 1＋或0.15-1.0g/24小时 | 2＋到3＋或>1.0-3.5g/24小时 | 4＋或3.5g/24小时 | 肾病综合征 | 死亡 |
| 疼痛 | | | | | | |
|  | | 分级 | | | | |
| 不良事件 | 简称 | 1 | 2 | 3 | 4 | 5 |
| 疼痛 | 疼痛 | 轻微疼痛，不影响功能 | 中度疼痛，疼痛或止疼药影响功能，但不影响ADL | 剧烈疼痛：疼痛或止痛药严重影响ADL | 致残 | － |
| 肺部/上呼吸道 | | | | | | |
| 肺纤维化（放射影像学改变） | 肺纤维化 | 轻微的放射影像学改变（或片状或双侧肺底改变），根据放射影像学表现，估计整个肺容积中发生纤维化的部分<25%. | 片状或双侧肺底改变，根据放射影像学表现，估计整个肺容积中发生纤维化的部分25－<50%. | 密集或广泛浸润/实变，根据放射影像学表现，估计整个肺容积中发生纤维化的部分50－<75%. | 根据放射影像学表现，估计整个肺容积中发生纤维化的部分≥75%;蜂窝状变化 | 死亡 |
| 备注：肺纤维化通常是放疗或多模式联合治疗（包括手术）3个月后发现的“晚期效应“。认为肺纤维化是疤痕/纤维化肺组织，与肺炎的鉴别较困难，后者通常见于放疗或多模式联合治疗后3个月内。  还要考虑：成人呼吸窘迫综合症（ARDS）；咳嗽；呼吸困难（气短）；缺氧；感染（有临床或微生物学记录）伴3或4级中性粒细胞（ANC<1.0×10^9^/L）-选择：感染伴正常ANC或1或2级中性粒细胞－选择：感染伴不明ANC－选择 | | | | | | |
| 综合征 | | | | | | |
|  | | 分级 | | | |  |
| 不良事件 | 简称 | 1 | 2 | 3 | 4 | 5 |
| 流感样综合征 | 流感样综合征 | 有症状，但不影响功能 | 中度或导致患者难以从事某些ADL | 严重症状，影响ADL | 致残 | 死亡 |
| 备注：流感样综合征代表多种症状的集合，包括伴卡他症状的咳嗽、发热、头疼、倦怠、鸡肉酸痛和虚弱，用于症状集合出现并伴有单一病理生理改变之时。 | | | | | | |
|  | | | | | | |
| 血管 | | | | | | |
| 静脉炎（包括浅表性血栓形成） | 静脉炎 | － | 存在 | － | － | － |
| 还要考虑：注射部位反应/外渗改变 | | | | | | |

**附录三：实体肿瘤的疗效评价标准1.1 版**

（Response Evaluation Criteria in Solid Tumors RECIST Version 1.1）

**1 肿瘤在基线水平的可测量性**

**1.1 定义**

在基线水平上，肿瘤病灶/淋巴结将按以下定义分为可测量和不可测量两种：

**可测量病灶**

肿瘤病灶：至少有一条可以精确测量的径线（记录为最大径），其最小长度如下：

- CT扫描 10 mm（CT扫描层厚不大于5mm）
- 临床常规检查仪器 10 mm（肿瘤病灶不能用测径仪器准确测量的应记录为不可测量）
- 胸部X-射线 20 mm
- 恶性淋巴结：病理学增大且可测量，单个淋巴结CT扫描短径须≥15 mm（CT扫描层厚推荐不超过5 mm）。基线和随访中，仅测量和随访短径

**不可测量病灶**

所有其他病灶，包括小病灶（最长径＜10 mm或者病理淋巴结短径≥10 mm至＜15 mm）和无法测量的病灶。无法测量的病灶包括：脑膜疾病、腹水、胸膜或者心包积液、炎性乳腺癌、皮肤/肺的癌性淋巴管炎、影像学不能确诊和随诊的腹部包块，以及囊性病变。

**关于病灶测量的特殊考虑**

骨病灶、囊性病灶和先前接受过局部治疗的病灶需要特别注明：

骨病灶：

- 骨扫描，PET扫描或者平片不适合于测量骨病灶，但是可用于确认骨病灶的存在或 者消失；
- 溶骨性病灶或者混合性溶骨/成骨病灶有确定的软组织成分，且软组织成分符合上述可测量性定义时，如果这些病灶可用断层影像技术如CT或者MRI进行评价，那么这些病灶可以作为可测量病灶；
- 成骨病灶属不可测量病灶。

囊性病灶：

- 符合放射影像学单纯囊肿定义标准的病灶，不应因其为定义上的单纯性囊肿，而认为是恶性病灶，既不属于可测量病灶，也不属于不可测量病灶；
- 若为囊性转移病灶，且符合上述可测量性定义的，可以作为是可测量病灶。但如果在同一病人中存在非囊性病灶，应优先选择非囊性病灶作为靶病灶。

局部治疗过的病灶：

- 位于曾放疗过或经其他局部区域性治疗的部位的病灶，一般作为不可测量病灶，除非该病灶出现明确进展。研究方案应详细描述这些病灶属于可测量病灶的条件。

**1.2 测量方法说明**

**病灶测量**

临床评价时，所有肿瘤测量都要以公制米制记录。所有关于肿瘤病灶大小的基线评定都应尽量在接近治疗开始前完成，且必须在治疗开始前的28天内（4周）完成。

**评价方法**

对病灶基线评估和后续测量应采用同样的技术和方法。除了不能用影像学检查，而仅能用临床检查来评价的病灶之外，所有病灶必须使用影像学检查进行评价。

临床病灶：临床病灶只有位于浅表且测量时直径≥10 mm时才能认为是可测量病灶（如皮肤结节等）。对于有皮肤病灶的患者，建议用含有标尺测量病灶大小的彩色照片作为存档。当病灶同时使用影像学和临床检查评价时，由于影像学更客观且研究结束时可重复审阅，应尽可能选用影像学评价。

胸部X片：当肿瘤进展作为重要研究终点时，应优先使用胸部CT，因为CT比X线更敏感，尤其对于新发病灶。胸部X片检测仅当被测量病灶边界清晰且肺部通气良好时适用。

CT、MRI：CT是目前用于疗效评价最好的可用可重复的方法。本指导原则对可测量性的定义建立在CT扫描层厚≤5 mm的基础上。如果CT层厚大于5 mm，可测量病灶最小应为层厚的2倍。MRI在部分情况下也可接受（如全身扫描）。

超声：超声不应作为一种测量方法用于测量病灶大小。超声检查因其操作依赖性，在测量结束后不具备可重复性，不能保证不同测量间技术和测量的同一性。如果在试验期间使用超声发现新病灶，应使用CT或者MRI进行确认。如果考虑到CT的放射线暴露，可以使用MRI代替。

内窥镜，腹腔镜检查：不建议使用这些技术用于肿瘤客观评价，但这种方法在取得的活检标本时可以用于确认CR，也可在研究终点为CR后复发或手术切除的试验中，用于确认复发。

肿瘤标志物：肿瘤标志物不能单独用来评价肿瘤客观缓解。但如果标志物水平在基线时超过正常值上限，用于评价完全缓解时必须回到正常水平。因为肿瘤标志物因病而异，在将测量标准写入方案中时需考虑到这个因素。有关CA-125缓解（复发性卵巢癌）及PSA（复发性前列腺癌）缓解的特定标准已经发表。且国际妇科癌症组织已制定了CA-125进展标准，即将被加入到卵巢癌一线治疗方案的肿瘤客观评价标准中。

细胞学/组织学技术：在方案规定的特定情况下，这些技术可用于鉴定PR和CR(如生殖细胞肿瘤的病灶中常存在残留的良性肿瘤组织)。当渗出可能是某种疗法潜在的副反应（如使用紫杉烷化合物或血管生成抑制剂的治疗），且可测量肿瘤符合缓解或疾病稳定标准时，在治疗过程中肿瘤相关的渗出出现或加重，可通过细胞学技术来确诊，以区分缓解（或疾病稳定）和疾病进展。

**2 肿瘤缓解的评估**

**2.1 靶病灶评估**

完全缓解（CR）：所有靶病灶消失，全部病理淋巴结（包括靶结节和非靶结节）短直径必须减少至＜10 mm。

部分缓解（PR）：靶病灶直径之和比基线水平减少至少30%。

疾病进展（PD）：以整个实验研究过程中所有测量的靶病灶直径之和的最小值为参照，直径和相对增加至少20%（如果基线测量值最小就以基线值为参照）；除此之外，必须满足直径和的绝对值增加至少5 mm（出现一个或多个新病灶也视为疾病进展）。

疾病稳定（SD）：靶病灶减小的程度没达到PR，增加的程度也没达到PD水平，介于两者之间，研究时可以直径之和的最小值作为参考。

**2.2 靶病灶评估的注意事项**

淋巴结：即使鉴定为靶病灶的淋巴结减小至10 mm以内，每次测量时仍需记录与基线对应的实际短直径的值（与基线测量时的解剖平面一致）。这意味着如果淋巴结属于靶病灶，即使达到完全缓解的标准，也不能说病灶已全部消失，因为正常淋巴结的短直径就定义为＜10 mm。在CRF表或其他的记录方式中需在特定位置专门记录靶淋巴节病灶：对于CR，所有淋巴节短直径必须＜10 mm；对于PR、SD和PD，靶淋巴节短直径实际测量值将被包含在靶病灶直径的和之中。

小到无法测量的靶病灶：临床研究中，基线记录过的所有病灶（结节或非结节）在后面的评估中都应再次记录实际测量值，即使病灶非常小（如2 mm）。但有时候可能太小导致CT扫描出的图像十分模糊，放射科医生也很难定义出确切的数值，就可能报告为“太小而测量不到”。出现这种情况时，在CRF表上记录上一个数值是十分重要的。如果放射科医生认为病灶可能消失了，那也应该记录为0 mm。如果病灶确实存在但比较模糊，无法给出精确的测量值时，可默认为5 mm。（注：淋巴结出现这种情况的可能性不大，因其正常情况下一般都具有可测量的尺寸，或者像在腹膜后腔中一样常常为脂肪组织所包绕；但是如果也出现这种无法给出测量值的情况，也默认为5 mm）。5 mm的默认值源于CT扫描的切割厚度（这个值不因CT不同的切割厚度值而改变）。由于同一测量值重复出现的几率不大，提供这个默认值将降低错误评估的风险。但需要重申的是，如果放射医生能给出病灶大小的确切数值，即使病灶直径小于5 mm，也必须记录实际值。

分离或结合的病灶：当非结节性病灶分裂成碎片状时，将各分离部分的最长径加起来计算病灶的直径之和。同样，对于结合型病灶，通过各结合部分间的平面可将其区分开来，然后计算各自的最大直径。但如果结合得密不可分，最长径应取融合病灶整体的最长径。

**2.3 非靶病灶的评估**

这部分对非靶病灶肿瘤的缓解标准进行了定义。虽然一些非靶病灶实际可测量，但无需测量，只需在方案规定的时间点进行定性评估即可。

完全缓解（CR）：所有非靶病灶消失，且肿瘤标记物恢复至正常水平。所有淋巴结为非病理尺寸（短径＜10 mm）。

非完全缓解/非疾病进展：存在一个或多个非靶病灶和/或持续存在肿瘤标记物水平超出正常水平。

疾病进展：已存在的非靶病灶出现明确进展。注：出现一个或多个新病灶也被视为疾病进展。

**2.4 关于的非靶病灶进展评估的特别注意事项**

关于非靶病灶进展的定义补充解释如下：当患者存在可测量非靶病灶时，即使靶病灶评估为稳定或部分缓解，要在非靶病灶的基础上作出明确进展的定义，必须满足非靶病灶整体的恶化程度已达到必须终止治疗的程度。而一个或多个非靶病灶尺寸的一般性增大往往不足以达到进展标准，因此，在靶病灶为稳定或部分缓解时，仅依靠非靶病灶的改变就能定义整体肿瘤进展的情况几乎是十分稀少的。

当患者的非靶病灶均不可测量时：在一些Ⅲ期试验中，当入选标准中没有规定必须存在可测量病灶时，就会出现这种情况。整体评估还是参照上文标准，但因为这种情况下没有病灶的可测量数据。非靶病灶的恶化不容易评估（根据定义：必须所有非靶病灶都确实无法测量），因此当非靶病灶改变导致整体疾病负荷增加的程度相当于靶病灶出现疾病进展时，依据非靶病灶作出明确进展的定义，需要建立一种有效的检测方法来进行评估。如描述为肿瘤负荷增加相当于体积额外增加73%（相当于可测量病灶直径增加20%）。又比如腹膜渗出从“微量”到“大量”；淋巴管病变从“局部”到“广泛播散”；或在方案中描述为“足够至改变治疗方法”。例子包括胸膜渗出液从痕量到大量，淋巴受累从原发部位向远处扩散，或者在方案中可能被描述为“有必要进行治疗方面的改变”。如果发现有明确的进展，该患者应该在那个时点总体上视为疾病进展。最好具有客观标准可适用于不可测量的病灶的评估，注意，增加的标准必须是可靠的。

**2.5 新病灶**

新的恶性病灶的出现预示着疾病的进展；因此针对新病变的一些评价是非常重要的。目前没有针对影像学检测病灶的具体标准，然而一种新的病灶的发现应该是明确的。比如说，进展不能归因于影像学技术的不同，成像形态的改变，或者肿瘤以外的其它病变（如：一些所谓新的骨病灶仅仅是原病灶的治愈，或原病灶的复发）。当病人的基线病灶出现部分或完全反应时，这一点非常重要的，例如：一例肝脏病灶的坏死可能在CT报告上定为新的囊性病变，而其实不是。

在随访中已检测到的而在基线检查中未发现的病灶将视为新的病灶，并提示疾病进展。例如一个在基线检查中发现有内脏病灶的患者，当他做CT或MRI的头颅检查时发现有转移灶，该患者的颅内转移病灶将被视为疾病进展的依据，即使他在基线检查时并未做头颅检查。

如果一个新的病灶是不明确的，比如因其形态小所致，则需要进一步的治疗和随访评价以确认其是否是一个新的病灶。如果重复的检查证实其是一个新的病灶，那么疾病进展的时间应从其最初的发现的时间算起。

病灶进行FDG-PET评估一般需要额外的检测进行补充确认，FDG-PET检查和补充CT检查结果相结合评价进展情况是合理的（尤其是新的可疑疾病）。新的病灶可通过FDG-PET检查予明确的，依据以下程序执行：

基线FDG-PET检查结果是阴性的，接下来随访的FDG-PET检查是阳性的，表明疾病的进展。

没有进行基线的FDG-PET检查，后续的FDG-PET检查结果是阳性的：

如果随访的FDG-PET阳性检查结果发现的新的病变灶与经CT检查结果相符，证明是疾病进展。

如果随访的FDG-PET的阳性检查结果发现的新的病变灶未能得到CT检查结果的确认，需再行CT检查予以确认（如果得到确认，疾病进展时间从前期FDG-PET检查发现异常算起）。

如果随访的FDG-PET的阳性检查结果与经CT检查已存在的病灶相符，而该病灶在影像学检测上无进展，则疾病无进展。

**2.6 评估缺失和不可评价说明**

如果在某个特定时间点上无法进行病灶成像或测量，则该患者在该时间点上无法评价。如果在一个评价中只能对部分病灶进行评价，通常这种情况视为在那个时间点无法评价，除非有证据证实缺失的病灶不会影响指定时间点的疗效反应评价。

**2.7 疗效评估的特别提示**

当结节性病灶被包括在总的靶病灶评估中，同时该结节大小缩小到“正常”大小时（＜10 mm），它们依然会有一个病灶大小扫描报告。为了避免过高评估基于结节大小增加所反映的情况，即便是结节正常，测量结果也将被记录。正如前面已经提及的，这就意味着疗效为完全缓解的受试者，CRF表上也不会记录为0。

若试验过程中需要进行疗效确认，重复的“不可测量”时间点将使最佳疗效评估变得复杂。试验的分析计划必须说明，在确定疗效时，这些缺失的数据/评估可以被解释清楚。比如，在大部分试验中，可以将某受试者PR-NE-PR的反应作为得到了疗效确认。

当受试者出现健康情况整体恶化要求停止给药治疗，但是没有客观证据证明时，应该被报道为症状性进展。即便在治疗终止后也应该尽量去评估客观进展的情况。症状性恶化不是客观反应的评估描述：它是停止治疗的原因。那样的受试者的客观反应情况将通过表1到3所示的目标和非目标病灶情况进行评估。

定义为早期进展，早期死亡和不可评估的情况是研究特例，且应该在每个方案中进行明确的描述（取决于治疗间期和治疗周期）。

在一些情况下，从正常组织中辨别局部病灶比较困难。当完全缓解的的评估基于这样的定义时，我们推荐在进行局部病灶完全缓解的疗效评估前进行活检。当一些受试者局部病灶影像学检测结果异常被认为是代表了病灶纤维化或者疤痕形成时，FDG-PET被当作与活检相似的评估标准，用来对完全缓解进行疗效确认。在此种情况下，应该在方案中对FDG-PET的应用进行前瞻性描述，同时以针对此情况专科医学文献的报告作为支持。但是必须意识到的是由于FDG-PET和活检本身的限制性（包括二者的分辨率和敏感性高低），将会导致完全缓解评估时的假阳性结果。

**表1 时间点反应：有靶病灶的受试者（包括或者不包括非靶病灶）**

| **目标病灶** | **非目标病灶** | **新病灶** | **总缓解** |
| --- | --- | --- | --- |
| CR | CR | 非 | CR |
| CR | 非CR/非PD | 非 | PR |
| CR | 不能评估 | 非 | PR |
| PR | 非进展或者不能完全评估 | 非 | PR |
| SD | 非进展或者不能完全评估 | 非 | SD |
| 不能完全评估 | 非进展 | 非 | NE |
| PD | 任何情况 | 是或否 | PD |
| 任何情况 | PD | 是或否 | PD |
| 任何情况 | 任何情况 | 是 | PD |
| CR=完全缓解 | PR=部分缓解 | SD=疾病稳定 | PD=疾病进展  NE=不能评估 |

**表2 时间点反应-仅有非目标病灶的受试者**

| **非目标病灶** | **新病灶** | **总缓解** |
| --- | --- | --- |
| CR | 非 | CR |
| 非CR或者非PD | 非 | 非CR或非PD |
| 不能完全评估 | 非 | 不能评估 |
| 不能明确的PD | 是或否 | PD |
| 任何情况 | 是 | PD |

注解：对于非目标病灶，“非CR/非PD”是指优于SD的疗效。由于SD越来越多作为评价疗效的终点指标，因而制定非CR/非PD的疗效，以针对未规定无病灶可测量的情况。

对于不明确的进展发现（如非常小的不确定的新病灶；原有病灶的囊性变或坏死病变）治疗可以持续到下一次评估。如果在下一次评估中，证实了疾病进展，进展日期应该是先前出现疑似进展的日期。

**表3 CR和PR疗效需要确认的最佳总缓解**

| **第一个时间点总缓解** | **随后时间点总缓解** | **最佳总缓解** |
| --- | --- | --- |
| CR | CR | CR |
| CR | PR | SD，PD或PR^a^ |
| CR | SD | 如果SD持续足够时间则为SD，否则应为PD |
| CR | PD | 如果SD持续足够时间则为SD，否则应为PD |
| CR | NE | 如果SD持续足够时间则为SD，否则应为NE |
| PR | CR | PR |
| PR | PR | PR |
| PR | SD | SD |
| PR | PD | 如果SD持续足够时间则为SD，否则应为PD |
| PR | NE | 如果SD持续足够时间则为SD，否则应为NE |
| NE | NE | NE |

注解：CR即是完全缓解，PR是部分缓解，SD是疾病稳定，PD是疾病进展，NE即不可评价。上标“a”：如果在第一个时间点CR真正出现，在随后的时间点出现的任何疾病，那么即便相对于基线该受试者疗效达到PR标准，其疗效评价在之后的时间点仍然为PD（因为在CR之后疾病将再次出现）。最佳缓解取决于是否在最短的治疗间隔内出现SD。然而有时第一次评价为CR，但随后的时间点扫描提示小病灶似乎依然出现，因而实际上受试者疗效在第一个时间点应该是PR而不是CR。在这种情况下，首次CR判断应该被修改为PR，同时最好的反应是PR。

**2.8. 疗效评估/缓解期的确认**

**疗效确认**

对于以肿瘤缓解疗效为主要研究终点的非随机临床研究，必须对PR和CR的疗效进行确认，以保证疗效不是评价失误的结果。以疾病稳定或者疾病进展为主要研究终点的研究中，不再需要疗效确认，因为这对于试验结果的解释没有价值。SD的情况下，在试验开始后的最短时间间隔内（一般不少于6~8周），至少有一次测量符合方案中规定的SD标准。

**总缓解期**

总缓解期是从测量首次符合CR或PR（无论哪个先测量到）标准的时间到首次真实记录疾病复发或进展的时间（把试验中记录的最小测量值作为疾病进展的参考）。总完全缓解时间是从测量首次符合CR标准的时间到首次真实记录疾病复发或进展的时间。

**疾病稳定期**

　　是从治疗开始到疾病进展的时间（在随机化试验中，从随机分组的时间开始），以试验中最小的总和作为参考（如果基线总和最小，则作为PD计算的参考）。疾病稳定期的临床相关性因不同研究和不同疾病而不同。如果在某一特定的试验中，以维持最短时间稳定期的病人比例作为研究终点，方案应特别说明SD定义中两个测量间的最短时间间隔。

注意：缓解期、稳定期以及PFS受基线评价后随访频率的影响。定义标准随访频率不属于本指导原则范围。随访频率应考虑许多因素，如疾病类型和分期、治疗周期及标准规范等。但若需进行试验间的比较，应考虑这些测量终点准确度的限制。

**2.9 PFS/TTP**

晚期肿瘤许多试验以PFS或者TTP作为主要研究终点。如果方案要求所有患者都有可测量病灶，进展评价就相对简单。越来越多的试验允许有可测量病灶和无可测量病灶的患者都可以进入试验。在这种情况下，必须对无可测量病灶患者疾病进展的临床发现进行详细明确的描述。因为进展日期常有确定偏差，各试验组的观测时间点安排应该相同。

**附录四：RECIST 1.0和RECIST 1.1版主要异同小结**

|  | RECIST 1.0 | RECIST 1.1 | 合理性 |
| --- | --- | --- | --- |
| 可测量病灶最小值 | 螺旋CT：10 mm；  非螺旋CT：20 mm  临床：20 mm | CT：10 mm；  未对螺旋/非螺旋作要求  临床：10 mm（必须为测径器可测量的病灶） | 大部分影像仪器的扫描厚度为5 mm或小于5 mm。在扫描层厚大于5 mm时，需提出更详细最小可测量病灶的说明；  临床病灶使用测径仪器可以实现这一目标 |
|  | 淋巴结未提及 | CT：:靶病灶短径≥15 mm；非靶病灶短径：≥10 mm-＜15 mm；非病理性病灶短径＜10 mm | 因为淋巴结是正常的结构，必须定义其病理性增大的情况。短径是最敏感的 |
| 特别提到的可测量病灶 | 无 | 特别提到骨病变和囊性病变 | 使以前常见问题得到更清晰解答 |
| 总体肿瘤负荷 | 10个病灶（每个器官5个） | 5个病灶（每个器官2个） | 数据库分析显示：病灶数从10降到5不会造成信息缺失，且每个器官最多2个病灶对每个病变部位已具有足够的代表性 |
| 靶病灶评价标准 | CR：未提及淋巴结  PD：在径和最小值基础上至少增加20%或出现新病灶 | CR：淋巴结短轴必须＜10 mm  PD：在径和最小值（如果基线病灶值最小则取基线病灶值）基础上至少增加20%,且绝对值至少增加5 mm,或出现新病灶 | 与正常淋巴结大小一致 ；  明确了如果基线测量值在所有测量值中为最小，当径和总值非常小且20%的增加值在误差范围内时，以基线值作为参考可确保PD的准确定义 |
| 非靶病灶评价标准 | PD：出现明确的进展 | 对PD的明确进展进行了更为详细地阐述，指出其衡量不应仅根据靶病灶或单个病灶的病情变化，而应考虑整体病情的进展情况 | 纠正了RESIST 1.0版本中的一些混乱概念，1.0版本中规定在一些情况下，即便靶病灶是稳定或缓解状态时，也将任何非靶病灶的增加视为PD |
| 新病灶 | 无 | 新病灶的新部位 | 为判断新病灶提供指导原则（继而定义PD) |
| 总体评价 | 对靶病灶和非靶病灶进行综合评价 | 分两部分进行评价：一部分对靶病症和非靶病症进行综合评价；另一部分单独评价非靶病灶特殊注意事项：如何评价和测量淋巴结；关于残留组织的CR评价；对疾病进展尚不明确的讨论 | 目前RESIST标准应用的临床试验中，以PFS为进展终点，且不是所有的患者在基线测量时均具有可测量病灶，分两部分评价更具合理性 |
| 疗效确认检测 | 对于CR和PR:应在首次疗效评价4周后予以确认 | 仅在主要目的是观察疾病缓解的非随机试验中保留采用此标准 | 资料显示：当不进行疗效确认时，缓解率提高；在在没设对照组和主要以观察缓解率为目标的临床试验中需要特别重视 |
| 无进展生存期 | 只是一般性评价 | 以PFS或无进展比例作为II期临床试验的研究目的，采用特殊的规定；在III期临床试验中则采用更具体的PFS疗效评价 | III期临床试验中对PFS的广泛应用，使得需要制订针对不可测量疾病患者PD评价的指导原则 |
| 疗效反应结果的报告 | 有9种关于报告II期临床试验结果的指导建议 | 将II期临床和III期临床试验分开，在III期临床试验中，建议将关于报告临床试验结果的9种指导减为5种 | 简化报告，阐明如何使II期临床和III期临床试验结果报告一致 |
| III期临床试验的疾病缓解 | 根据具体方案的规定，按照更加灵活的标准说明 | II期临床和III期临床采用相同的标准 | 在随机试验中当疾病缓解不是主要研究目的时，可以通过减少观察病灶的数量和取消相关确认的要求而简化疗效评价，从而不需要制订区分的评价原则 |

**附录五—根据体表面积计算卡培他滨使用剂量**

卡培他滨起始剂量1000 mg/m^2^，下表描述了根据体表面积计算的标准和减少的剂量：以下表格仅适用于500 mg规格的片剂，且100%的剂量水平指1000 mg/m^2^每日两次（单日剂量2000 mg/m^2^）的剂量水平。

| **100%的剂量水平**  **= 1000 mg/m^2^每日两次给药** | | 所服用的500 mg规格的药片数量 | |
| --- | --- | --- | --- |
| 体表面积（m^2^） | 每次给药的平均剂量（mg） | 早晨 | 晚间 |
| < 1.12 | 1000 | 2 | 2 |
| 1.13 – 1.37 | 1250 | 2 | 3 |
| 1.38 – 1.62 | 1500 | 3 | 3 |
| 1.63 – 1.87 | 1750 | 3 | 4 |
| > 1.88 | 2000 | 4 | 4 |

| **75%的剂量水平** | | 所服用的500 mg规格的药片数量 | |
| --- | --- | --- | --- |
| 体表面积（m^2^） | 每次给药的平均剂量（mg） | 早晨 | 晚间 |
| < 1.12 | 750 | 1 | 2 |
| 1.13 – 1.37 | 1000 | 2 | 2 |
| 1.38 – 1.87 | 1250 | 2 | 3 |
| > 1.88 | 1500 | 3 | 3 |

| **50%的剂量水平** | | 所服用的500 mg规格的药片数量 | |
| --- | --- | --- | --- |
| 体表面积（m^2^） | 每次给药的平均剂量（mg） | 早晨 | 晚间 |
| < 1.12 | 500 | 1 | 1 |
| 1.13 – 1.87 | 750 | 1 | 2 |
| > 1.88 | 1000 | 2 | 2 |

**卡培他滨的治疗中断和休息期延长**

**正常治疗：**

第１天

第８天

第１５天

第２２天

第２９天

第３６天

第４３天

第５０天

**治疗期间中断：**

中断的含义是停止了当天的治疗。原定的治疗计划仍应继续进行。

**休息期延长：**

若由于毒性导致休息期延长，则之后的治疗周期仍应保持完整。

１４天

１４天

７天

**附录六：万特普安®（铜绿假单胞菌注射液）说明书**

**
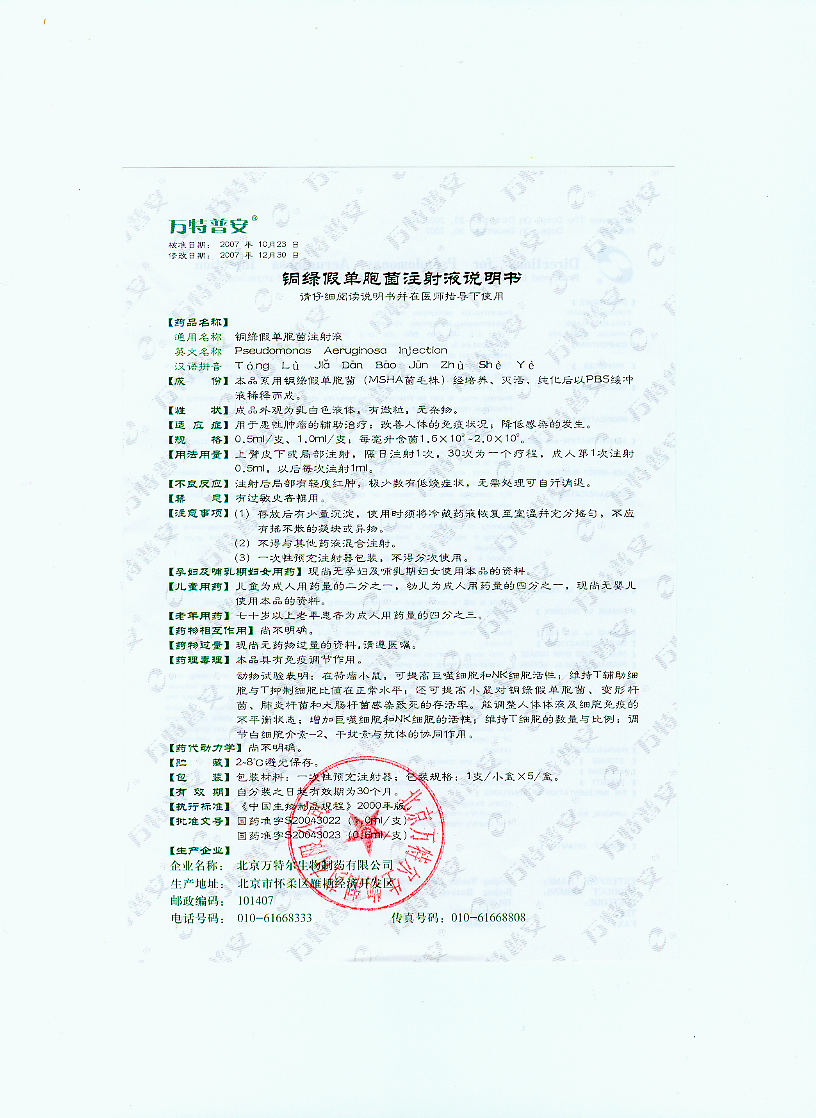
**
