# Supplementary material for: Phase II Study of Pseudomonas aeruginosa-Mannose-Sensitive Hemagglutinin in Combination with Capecitabine for Her-2–Negative Metastatic Breast Cancer Pretreated with Anthracycline and Taxane
Source: PLoS One. 2015 Mar 13;10(3):e0118607. doi: 10.1371/journal.pone.0118607 (PMC4359133; doi:10.1371/journal.pone.0118607)
Supplement: S2 Protocol — (DOC) [file pone.0118607.s003.doc]

**Phase II study of *Pseudomonas aeruginosa*-mannose-sensitive hemagglutinin in combination with capecitabine for Her-2–negative metastatic breast cancer**

|  |  |
| --- | --- |
|  |  |

**Date final：2011/02/01**

**version：1.0**

**Protocol summary**

| Ttitle | Phase II study of *Pseudomonas aeruginosa*-mannose-sensitive hemagglutinin(PA-MSHA) in combination with capecitabine for Her-2–negative metastatic breast cancer |
| --- | --- |
| Study site | Fudan university cancer centre |
| Study objectives | To assess the efficacy and safety of PA-MSHA in combination with capecitabine in Her-2 negative metastatic breast cancer. |
| Study design | Phase II, single arm, single center clinical trial. |
| Study population | MBC pretreated with antracycline and taxane. |
| Number of patients | 96 |
| Study regime | PA-MSHA 1 mg was administered subcutaneously every other day and capecitabine1000 mg/m^2^ orally twice a day for 2 weeks on, 1 week off. |
| Primary endpoint | Progression free survival（PFS） |
| Secondary endpoint | Overall response rate（ORR）、Overall survival（OS）、safety、exploratory endpoints such as immune index. |
| Inclusion criteria | 1) Women aged between 18 and 70 years；  2) Histologically confirmed Her-2–negative MBC；  3) Not suitable for local treatment;  4) Pretreated with antracyclene and taxane；   1. Prior capecitabine-containing therapy was permitted if the disease had responded to the drug previously and progressed at least 4 months after drug discontinuation； 2. ECOG performance status of no more than 2；   7) Adequate organ function；  8) Having the following laboratory values; WBC≥4×10^9^/L；Hb≥90 g/L；plt≥100×10^9^/L；  9) Written informed consent should be obtained before study procedure. |

**I．Introduction**

1. **Treatment status of metastatic breast cancer (MBC)**

Breast cancer is the most common malignant tumor in women. There are about 1 million new breast cancer patients in the world every year, and more than 410 thousands died of it. The incidence of breast cancer also rises fast in recent years in China. In 2000, the age-adjusted prevalence of breast cancer was 19.9/100 thousands in China. And it has become the most common malignant tumor in Chinese women. Though significant progress has been made in the diagnosis and treatment of breast cancer, most of the breast cancer patients still appear recurrence eventually due to the biological behaviors of the cancer as well as the lower early diagnosis rate. Metastatic breast cancer (MBC) has been considered as an incurable disease currently, with the median survival time of only 2-3 years. MBC treatment aims at prolonging the survival time, reducing the symptoms associated with the disease and improving the patient’s quality of life. Therefore, it is particularly important to explore new antitumor drugs and new treatment, reduce the side effect of treatment and improve the patient’s quality of life.

Recent studies have shown that invasive breast cancer can be divided into several basic subtypes with cDNA microarray, including luminal A, luminal B and hormone receptor negative subtype, namely the HER2 positive and basal- like subtypes. In the conventional treatment, the patients were classified according to whether the patients had excessive expression of hormone receptor (estrogen receptors (ER) and/or progesterone receptors (PgR)) and/or human epidermal growth factor receptor-2 (HER-2). For hormone receptor-positive tumors, endocrinotherapy is the common selection due to relatively good response, easy to use and low toxicity. For HER2 positive subtype, Herceptin based therapies were often chosen. For three negative patients or patients with hormone receptor-positive but also with drug resistance against endocrinotherapy, cytotoxic chemotherapy is the standard treatment. Monotherapy and combination treatment are both effective MBC treatments. Combination therapy has improved disease remission rate and time of disease progression, but it has little benefit on survival, and in addition, the toxicity was increased remarkably because of administration of a variety of cytotoxic drugs.

Commonly used chemotherapy drugs in MBC treatments include anthracyclines (e.g., doxorubicin (DXR), epirubicin (EPI)), taxanes (e.g., docetaxel, paclitaxel), alkylating agent (such as cyclophosphamide), capecitabine (Xeloda), vinocrelbine and gemcitabine ^[1]^. Anthracyclines are considered to be one of the most effective treatments for breast cancer, which are usually used in the initial treatment. Taxanes have also been considered a very effective agent for MBC ^[2]^. But as anthracyclines and taxanes gradually widely applied in the adjuvant and neoadjuvant therapy, drug selection for MBC is limited ^[3]^.

For patients pretreated with anthracyclines and taxanes, capecitabine can be an important choice, which is also the only drug approved to use in this setting. Patients pretreated with anthracyclines and taxanes have a response rate as high as 28% in monotherapy with capecitabine ^[4-6]^. The drug also has the advantage of oral administration and mild bone marrow suppression. For patients pretreated with anthracyclines and taxanes, other choices include gemcitabine, vinocrelbine, liposomes doxorubicin ^[7]^.

In addition, biological treatment has gradually entered into the clinical application of metastatic breast cancer treatment ^[1]^. For HER-2 positive tumor patients, trastuzumab has become the key agent in standard therapy of this subgroup. However, for HER-2 negative breast cancer, there is no standard biological treatment . Bevacizumab, the anti-angiogenesis drug which used to be a hot spot, has come to be questioned in MBC treatment now.

**2. Capecitabine and MBC treatment**

Some clinical studies have evaluated the efficacy of capecitabine used alone or in combination for MBC. Capecitabine combined with docetaxel has been approved for MBC patients pretreated with anthracycline-based chemotherapy [8]. In addition, capecitabine is currently approved as monotherapy for MBC patients pretreated with taxane and anthracycline-based chemotherapy. Capecitabine is administered at the dose of 1250 mg/m^2^, twice every day, for 2 weeks on and 1 week off.

Some previous phase II clinical studies show effectiveness of capecitabine as monotherapy in patients pretreated with taxane or anthracycline-based chemotherapy ^[4,5,9,10]^. In these studies, capecitabine monotherapy has a response rate of 15-28%, with the median progression-free survival (PFS) of 3.1-4.9 months. Recently, a number of phase III studies also well expound the efficacy and safety of capecitabine in monotherapy or combination therapy.

A large phase III clinical trial has compared the effect and safety of capecitabine used alone or combined with Ixabepilone in 752 cases of patients pretreated with taxane or anthracyclines ^[11]^. Median PFS is 4.2 months in monotherapy of capecitabine, with the response rate of 14%. Though median PFS and response rate are increased in the combination therapy group (5.8 months and 35%), the side effects are also significantly increased. Incidence of grade 3 or 4 sensory neuropathy, malaise and leukopenia are 21%, 9% and 68% in the combination group, and in the monotherapy group, the corresponding rates are 0%, 3% and 11%。

There is another famous phase III clinical trial – the RIBBON-2 study enrolled 462 patients who were pretreated with taxane or anthracyclines ^[12]^. Patients were randomly assigned to monotherapy group (capecitabine) or combination (capecitabine combined with bevacizumab) therapy group. The results show that the response rate was higher in combination group (19.8% vs 9.1%), but there was no improvement for PFS and overall survival (OS).

All clinical data have showed that capecitabine as monotherapy has good safety profile ^[13]^. Hand - foot syndrome is the most common adverse events, which is not life-threatening, and can be well controlled via dose reduction. Capecitabine can also cause mild to moderate gastrointestinal adverse events (such as diarrhea, stomatitis). When the dosage of capecitabine is reduced to 1000 mg/m^2^ twice a day, grade 3/4 diarrhea incidence will be lower than those of the standard therapy (1250 mg/m^2^ twice a day) ^[14]^. Importantly, the effective is not compromised ^[5]^.

Capecitabine is also used in combination with biological agents such as Herceptin or bevacizumab. But in HER-2 negative patients, it has not obtained the expected effect in the combination therapy with bevacizumab. Therefore, the drug combination of capecitabine with other effective low-toxic drugs is still a good research orientation.

**3. PA-MSHA and its application in breast cancer**

Pseudomonas aeruginosa injection is a new kind of bacteria agent by genetic engineering preparation. The ingredient is “pseudomonas aeruginosa - mannose sensitive hemagglutination” (PA-MSHA) with independent intellectual property rights. Pseudomonas aeruginosa injection was approved by sFDA in 1998. It has been widely used in complementary therapy of a variety of malignancies, including breast cancer. Clinical data showed PA-MSHA can significantly inhibit the growth of tumors and improve life-quality in tumor patients ^[15,16]^.

**Action mechanism of** **PA-MSHA**

In 2009, Zhimin Shao et al (*Fudan University Shanghai Cancer Center*) has performed study on mechanism of pseudomonas aeruginosa injection to treat breast cancer. They have found that Pseudomonas aeruginosa injection has direct targeted killing effects on breast cancer cells (MDA-MB-231HM and MDA-MB-468 cells) in vitro and in vivo. Its unique MSHA fimbria can be specifically bound with mannose molecular of EGFR receptor on the surface of breast cancer cells, so as to block the activation of EGFR receptor and its intracellular signaling molecules (AKT, ERK), thus inhibiting the growth of breast cancer cells and inducing apoptosis; besides, Pseudomonas aeruginosa injection also can significantly reduce the levels of the proteins (MMP, E-Cadherin, VEGF) generated by breast cancer cells and associated with tumor metastasis and angiogenesis; The growth of breast cancer in nude mouse models have been effectively controlled, in addition, pulmonary metastasis rate and the number of pulmonary metastases are also significantly lower than the control group. The research outcomes were already published in *the Journal of Cellular Biochemistry* in 2009, and in *Oncogene* in 2010, which provides sufficient theoretical basis for pseudomonas aeruginosa injection to treat breast cancer in clinic ^[17,18]^.

On the other hand, pseudomonas aeruginosa injection is also found a highly efficient immune activation agent. According to a study in 2008, the MSHA fimbriae of pseudomonas aeruginosa injection can fully active the Toll-like receptors (TLR4) on the surface of immune cells ^[19]^; induce mononuclear cells (dendritic cells, macrophages) to differentiation and maturation; further active T lymphocytes (CTL) and the NK cells; induce specific and non-specific anti-tumor immune response; help the body to rebuild tumor immune surveillance and improve the microenvironment of tumor cells ^[20]^. Therefore, pseudomonas aeruginosa injection is a new kind of anti-tumor drug with dual mechanisms: “targeted therapy” and “immune therapy”. Pseudomonas aeruginosa injection-based combination chemotherapy can not only play its strength of targeted therapy, but also can improve the low immunity caused by chemotherapy, thus improving the sensitivity of tumor cells to chemotherapy and reducing the side effects of chemotherapy.

**Application of pseudomonas aeruginosa injection in breast cancer therapy**

Most of the researches on pseudomonas aeruginosa injection are about its application in neoadjuvant chemotherapy. In a single-center clinical study conducted by *Tianjin Medical University Cancer Hospital* in 2009, 40 women with locally advanced breast cancer (LABC) (II~III) were randomly divided into two groups. The experimental group were given TE chemotherapy combined with subcutaneous injection of pseudomonas aeruginosa injection, while in control group, only TE chemotherapy was adopted. Both of the groups were given radical mastectomy or breast-conserving surgery after 4 months of chemotherapy. The results show that the total response rate (RR) was 85% in experimental group, significantly higher than the control group by 60% (P<0.05); Pathologic complete response rate (pCR) was 30% in the experimental group, higher than the control group by 10%. For the patients’ physical condition, after the treatment, Karnofsky score in experimental group was obviously higher than the baseline and also higher than that of the control group (P<0.01). Serum IFN-γ and IL-2 were increased while serum IL-4 and IL-10 were reduced in the experimental group after the treatment, the indexes before and after the treatment were all significantly different (P<0.05); after the treatment, the above indexes are of statistically significant differences between the experimental group and the control group (P<0.01). For tumor cell apoptosis and metastasis associated protein, serum Caspase-3 was raised while serum VEGF, MMP-2 and MMP-9 were reduced in the experimental group after treatment (P<0.05). Safety evaluation showed that no severe extramedullary adverse reaction and the acute cardiac toxicity were observed in both the experimental group and control group ^[21]^.

In a single-center clinical study conducted by *The Second Xiangya Hospital of Central South University* in 2009, 60 women with LABC (T2~T4) were randomly divided into two groups. The experimental group were given TAC chemotherapy combined with subcutaneous injection of pseudomonas aeruginosa injection for 21 days (once every day); While in control group, only TAC chemotherapy was adopted, the patients were given chemotherapy for 2~4 cycles based on the curative effect, and then given surgery. The research results showed that after 2 cycles of chemotherapy, total RR was 76.7% in the experimental group, which was significantly higher than that of the control group. And after chemotherapy of 3~4 cycles, there were still significant differences. For the patients’ physical condition, Karnofsky score showed no significant difference before and after treatment in the experimental group, but in the control group, Karnofsky score was obviously reduced. For chemotherapy toxicity, the most common toxic effects in the two groups were nausea, vomiting, bone marrow suppression and alopecia. Whether the incidences or the degrees of toxic reaction were significantly decreased in the experimental group compared with the control group ^[22]^.

Breast cancer is a heterogeneous disease, where different subtypes have different sensitivities to chemotherapy as well as different prognosis. Patients with HER-2 receptor negative breast cancer, especially with Luminal A type breast cancer of HER-2 negative and ER positive, pCR rate that the neoadjuvant chemotherapy can achieve is lowest; Even for patients with three negative breast cancer, whose pCR rate is relatively high, the long-term prognosis is not ideal. For example, in the neoadjuvant chemotherapy study (Paclitaxel + Carboplatin (PCb), every week) of *Fudan University Shanghai Cancer Center*, pCR rates were higher in patients with ER negative, PR negative, HER-2 negative or three negative, the corresponding rates were 32.6%, 30.6%, 33.3% and 33.3%, while in patients with ER positive or Luminal A type (ER positive, HER-2 negative) breast cancer, the pCR rates were lower, of only 9.7% and 8.3%. Therefore, the patients must be give corresponding treatments according to the different types of biological behaviors ^[23]^. In general, for breast cancer patients with HER-2 receptor negative, the poor effect of the new adjuvant chemotherapy and Herceptin unavailable to use, further restrict the clinical treatment and therapeutic effect on such patients. So HER-2 receptor negative breast cancer is the difficulty in the treatment, which urgently needs new therapies.

On the basis of these encouraging preclinical and clinical data, we performed a single arm, phase II clinical trial using PA-MSHA combined with capecitabine as salvage treatment for Her-2–negative MBC patients pretreated with anthracyclines and taxanes.

**II.Study design**

This phase II, single centre, single arm, open-label study is designed to evaluate the efficacy and safety of capecitabine plus PA-MSHA in subjects with Her-2 negative metastatic breast cancer pretreated with anthracycline and taxane.

**III. Inclusion and exclusion criteria**

**1. Inclusion criteria**

1) Women aged between 18 and 70 years；

2)Histologically confirmed Her-2–negative MBC；

3) Not suitable for local treatment;

4) Pretreated with antracyclene and taxane；

5)Prior capecitabine-containing therapy was permitted if the disease had responded to the drug previously and progressed at least 4 months after drug discontinuation；

6)ECOG performance status of no more than 2；

7) Adequate organ function；

8) Having the following laboratory values; WBC≥4×10^9^/L；Hb≥90 g/L；plt≥100×10^9^/L；

9) Written informed consent should be obtained before study procedure.

**2. Exclusion criteria**

1. Pregnant or lactating females；
2. Prior history of malignancies, other than breast cancer；
3. Having participated in a prior investigational study within 1 months prior to Day 1 dosing；
4. Uncontrolled intercurrent illness；
5. Known sensitivity or allergy to PA-MSHA；
6. Unwilling or unable to comply the protocol；
7. Any other condition that investigator think the subject is not suitable for the study.

**IV.Treatment**

PA-MSHA 1 mg was administered subcutaneously every other day and capecitabine 1000 mg/m^2^ orally twice a day for 2 weeks on, 1 week off. The patient may continue treatment until unacceptable toxicities, disease progression.

**V. Assessment types**

**1 Efficacy assessment**

The response to treatment should be assessed by investigator based on RECIST version1.1 every two cycles.

**2. Safety assessment**

Safety will be monitored by assessing physical examination, performance status and laboratory examinations. Adverse events will be recorded based on CTC-AE version 3.0.

**VI Study procedure**

**1. Screening**

1. Patients information collection included Her-2, ER, PR status, Demography, Diagnosis and extent of cancer, relevant medical history, prior anti-cancer treatment；
2. Physical examination：including vital signs；
3. Hematology laboratory assessment（RBC、WBC、PLT、Hb）
4. Urinalysis（PRO、WBC、RBC）
5. ECG
6. Chemistry laboratory assessment（TP、TBIL、ALT、AST、AKP、A/G、LDH、 BUN、Cr）
7. Tumor assessment：chest CT、abdominal MRI、bone scan, ect.
8. Immunology assessment（CD3、CD4/CD8、NK、IL-2、IFN-γ、IL-4、IL-10 MMP-9、VEGF、E-Cadherin）
9. ECOG performance status.

**2. During treatment**

Laboratory assessment, physical examination and ECOG performance status will be assessed at the beginning of every cycle.

Chest CT、abdominal MRI and CEA、CA15-3、CA125 will be done every two cycles.

**VII. Concomitant medications and procedures**

**1. Hormonal therapy**

Concomitant hormonal therapy is not permitted.

**2. Biphosphonates**

Concomitant bisphosphonates is permitted if renal function is normal.

**3. Palliative radiation therapy**

Palliative radiation therapy in aim of relieving pain is permitted.

**4. Supportive care**

Supportive care including G-CSF, platelet transfusion and IL-11 is permitted.

All the concomitant drugs should be recorded in CRF.

**VIII. Study endpoints**

**1. primary endpoint：**progression free survival（PFS）

**2. 次要终点：**objective response rate（ORR）、overall survival（OS）、safety、immune index

**IX Usage method and precaution**

**Xeloda**

Drug administration are allowed to delay for 3 weeks in order to allow the toxicity to recover to baseline levels or grade 0~1. However, the dose of capecitabine should be reduced only in the event of severe toxic effects, including: adverse events ≥2 grade 2, such as diarrhea, persistent nausea/vomiting though given preventive measures, hand-foot syndrome (HFS), or elevated liver enzymes (AST and ALT > 2.5×ULN).

Once the patients decrease the dose of capecitabine, the dose will not be increased in the later cycles. But if the administration is interrupted and causes the treatment absence, the later treatment should continue to follow the schedule as planned, and the dose missing due to interruption of treatment should be made up. In addition, if the rest period extended over the next treatment cycle, capecitabine should be given a full dose of 14 days at restart of treatment and the treatment schedule shall be carried out as planned.

Please refer to appendix 5 for the standard dose according to body surface area and dose reduction of capecitabine.

Administration instructions

- Capecitabine of 1000 mg/m^2^ is given twice every day, from the first day to the 14th day in the cycle of 3 weeks.
- During administration, the patients should be instructed to swallow tablets with water within 30 minutes after meals (breakfast and dinner)
- Daily dose of capecitabine is determined according to the body surface area (BSA), please refer to appendix 5 for the right dose and the number of pills.

Pseudomonas aeruginosa injection (PA-MSHA)

- Manufacturer: Beijing Wanter Bio-pharmaceutical Co., LTD.;
- Properties: milky white liquid, with particles, without sundry;
- Specification: one-time prefilled syringes, with 1 ml/per syringe;
- Storage: 2~8℃ in the absence of light.
- Usage: subcutaneous injection, first dosage of 0.5 ml, then 1 ml/time, every other day (qod).

Precautions:

1) People with a history of allergy should cautiously use it, especially the crowd with a history of multiple drug allergy, who are not suggest to enter the group.

2) A small amount of precipitation may be seen during storage. The refrigerated liquid should be returned to room temperature and fully shaken before using. No block substance or foreign body should be seen.

3) Should not be mixed with other drug. Subcutaneous injection only; intracutaneous injection can cause serious local reactions; injection speed should be as slow as possible.

4) Packed with one-time prefilled syringe; should not use it for several times.

5) After subcutaneous injection, a few of patients may appear the phenomenon such as swelling, and pain, which can be recovered by themselves and do not need special treatment. The second injection should be in the other side. If subcutaneous nodule is seen or patients with severe symptoms, local hot compress is advised to promote drug absorption. Periumbilical and health upper limb can be used for subcutaneous injection in turn.

6) Resting more, drinking more water and keeping warm can reduce adverse reaction. For example, if excluded other causes, fever over 38.5 ℃ can be treated symptomatically, especially physical cooling method. With antipyretic analgesics agents (e.g., indomethacin suppository or emplastrum) the symptomatic treatment can be alleviated quickly.

7) If there is intolerant toxicity, the medication can be reduced to 2 times per week.

8) If the patients appear side effects, such as high fever and chills, clinical nursing should be strengthened, and the specific methods should be proposed by doctors according to the clinical experiences. Antipyretic analgesics agents are suggested to prevent the side effects in advance.

**X. Study suspension**

Study suspension refers to the clinical trials that are not over according to the plan stop due to the following reasons. The main purpose is to protect the rights and interests of the subjects, ensure the test quality, and avoid unnecessary economic losses.

- Disease progression;
- Unacceptable adverse events;
- Dose adjustment for more than twice;
- Investigator think it necessary to stop the test of the patients;
- The patients withdraw inform consent;
- The patients delay the treatment for more than 3 weeks;
- Death.

**XI. Process and report of adverse events**

**1. Adverse events**

Adverse events (AEs) refer to the adverse medical events occurred in patient or clinical trial subjects after using a drug, but they may not necessarily have causal relationship with the treatment.

**2. Reporting period of adverse events**

Adverse events occurring between randomization and the last follow-up, no matter related to the research drug or not, should be filled in the case report form. In addition, any adverse incident happens after the reporting period, if being estimated associated with the research drug, the researcher also should report it as adverse events.

**3. Serious adverse event (SAE)**

Adverse events in accordance with one or more of the following criteria are defined as serious adverse events:

- Death;
- Life-threatening;
- Events that may result in hospitalization or prolong the hospital stay
- Permanent or serious disability;
- Congenital malformations/defect, important medical events.

**4. Report pathways of serious adverse events**

For all serious adverse events, the patients must stop study procedure immediately, and should be given corresponding protection measures. Researchers must report the sponsor the serious adverse events within 24h on telephone or fax. The researchers should track the serious adverse event until it is solved. Relevant medical documents shall be recorded in the original file, including laboratory examination results report (such as X-ray, ECG, etc.).

**5. Records and reports**

For the adverse events during the test, onset time, symptoms, degree, duration, treatment measures and outcome, etc., should be recorded in case report form. And their relevance to test drugs should be evaluated, and recorded by the researchers in detail. The researcher should sign and date the report. Severity of adverse events will be classified according to the NCI CTC3.0 version. For each symptom, it should be reported the highest classification of the adverse events since the last follow-up.

Relationship between adverse events and the experimental drugs

|  | Certainly relevant | Very possible to be relevant | May be relevant | Suspect | Impossible to be relevant |
| --- | --- | --- | --- | --- | --- |
| With the reasonable time sequence after medication | + | + | + | + | — |
| Known reaction types | + | + | + | — | — |
| Effect relieved or disappeared after removing the drugs | + | + | ± | ± | — |
| Effects appear again when administrate the drugs again | + | ？ | ？ | ？ | — |
| Cannot explain with the patients’ disease | + | + | — | ± | — |

**XII. Sample size**

It is expected that the PFS will be prolonged from 4.2 months with monotherapy of Xeloda to 5.7 months after combined with pseudomonas aeruginosa injection. So according to a=0.05 and power of 85%, the needed sample size is 88 cases; 96 patients were required in consideration of 10 % expulsion rate.

**XIII. Data processing**

**1. Requirement of researchers to fill data**

1) All of the patients who have signed an informed consent form and have been included in the experiment through screening, should record all of the items of the case report form carefully and in detail, no blank or omission is allowed (fill a cross in the blank space without any record);

2) All of the data in case report form should be checked with the medical data of the patients, to ensure flawless;

3) Case report form is used as original data, so any correction can only made by a line and marginal note of the new data, sign of the researchers and the data;

5) Data significantly higher or beyond the acceptable range in clinical must be verified, and explained by researchers;

**2. Requirement of CRA (clinical researcher associate) to monitor data**

1) CRA should regularly examine whether the subjects of each test center are informed and agreed and the screening and inclusion situations;

2) To confirm the case report form is filled correctly and is consistent with the original data;

3) All the errors or omissions are correct or noted, signed and dated by the researchers;

4) Dose change, treatment changes, drug combination, periodic disease, etc. of each subject, need to be identified and recorded;

5) To verify the selected participants who drop out from the test or are failed to follow up, and the situations should be given in the case report forms;

6) To confirm all the adverse events are recorded, and the serious adverse events are recorded are made a report;

7) To confirm whether the drugs are supplied, stored, distributed, returned and recorded in accordance with the relevant provision;

8) Each selected case is required to complete the CRF. And the original completed CRF belongs to Beijing Wanter Bio-pharmaceutical Co., LTD. The original completed CRF should not be provided to the third parties in any form without the written consent of the company, unless the requirements from State Drug Administration (SFDA);

9) CRF is filled by the researchers, and each selected cases must complete the CRF. The finished CRF should be examined by the CRA. The first page is transferred to the data statistics unit for data entry and management work; the second to the sponsor to be archived; and the first page is given to the clinical pharmacology base of each center to be archived.

**3. Traceability of the data, filling and transfer of the CRF**

The original records are medical records of the research and need to be properly kept. Case report form is from the medical records, and is filled by the researchers. Each selected cases must complete the CRF. The finished CRF should be examined by the CRA. The first page is transferred to the data statistics unit for data entry and management work; after transfer, the CRF cannot be corrected.

**4. Data entry and correction**

Data administrators of the statistical unit are in charge of the data entry and management. Epidata software is adopted to establish the special database for data entry and management. In order to guarantee the accuracy of the data, double entry and correction should be conducted by two data administrators independently.

For questions exist in the CRFs, data administrator will fill out the DRQ (data ready queue), and ask the researchers through CRF. The researchers should answer as soon as possible. Then data administrators would modify the data according to the researcher’s answer, confirm and entry the data. They are allowed to send DRQ again if necessary.

**5. Data locking**

When the data are reviewed and the established database is confirmed to be correct. The data will be locked by the principal investigator, the sponsor, statistical analysts and the pharmaceutical supervisory mangers. After locking the data file cannot be changed again. Problems found after locking, can be revised in the statistical analysis program after confirmation.

**XIV Statistical analysis**

Professional statisticians are responsible for statistical analysis, and participate in the whole process from test design, implementation to the analysis and summary. Statistical analysis plan is established after the completion of test protocol and case report form, and is made necessary changes in the test process according to the needs. Statistical analysis plans should be finished before data locking. And after the statistical data analysis is completed, statistical analysis report is provided.

1. **Data set analsis**

**1) FUll Analysis Set-FAS**: refers to the sets of eligible cases and off cases, but does not include excluded cases.

2) **Per-Protocol population** (PP): refers to the sets of cases that meet eligibility criteria and complete the treatment, namely the cases that are in accordance with the test protocol and with good adherence, don’t use banned drugs, and complete the CRF.

3) **Safety set:** the cases at least receive one time of treatment.

**2. Statistical methods**

**Statistical description**

1) Whether it is in accordance with normal distribution; if not, modify the statistical method or transfer the data;

2) Whether there is outlier: With statistical and special analysis to determine to use or not

3) Whether there is any missing value: when one of the main curative effect indexes is not found in individual subjects, switch of the last observation data is made.

4) The proportion of off patients: should not be greater than 20%, otherwise should be analyzed;

5) Descriptive statistics analysis: point out the mean, standard deviation, maximum, minimum, credibility interval, credibility rate, etc.

**3. Analysis method**

1) Measurement data: t test, paired t test, rank-sum test, paired rank-sum test and other methods;

2) Enumeration data: Fisher’s exact test, etc.; rank-sum test is adopted for ranked data.

3) Curative effect analysis: CMH chi-square test or logistic regression is used for enumeration data; variance analysis or rank-sum test is adopted for measurement data according to the characteristics of data; Kaplan-meier method or Cos regression is adopted for survival data.

4) FAS analysis and PP analysis: the two analyses are performed at the same time for the main curative effect indexes.

**4. Statistical expression**

Bilateral inspection is adopted generally, P less than or equal to 0.05 will be considered a statistically significant discrimination.

**5. Statistical software**

SPSS software is used for analysis.

**6. Test type**

Superiority test

**7. Content of statistical analysis**

1) Distribution of the two groups of cases: total off rate and the off rate due to adverse events in the two groups are compared with chi-square test.

2) Comparable analysis: demographic data and other basic indexes are compared to measure the comparability of the two groups.

3) Compliance analysis: to compare the two groups of patients that whether they are on a stricter medication regime, or use drugs and food forbidden in the regime.

4) Validity analysis: the main index and overall index are analyzed with PP and FAS analysis; because this study is a multicenter clinical trial, during the analysis, the influences of center effect on the curative index should be considered.

5) Security analysis: first, according to the requirements of adverse reaction correlation, adverse events and adverse reactions of the two groups should be listed, including the number of adverse events, cases of laboratory examination indexes turned from normal to abnormal before and after the test and different rate). The chi-square test is adopted to analyze adverse reactions.

**XV. Quality control and assurance**

During the test, the test centers will be monitored and visited regularly to ensure the test scheme carried out. The original data should be checked to confirm the consistency of the data on CRF.

**XVI. Ethical norm**

This research must be in strict accordance with the requirements of SFDA GCP and the declaration of Helsinki.

**Independent ethics committee (IEC)**

Before the start of the clinical research, test plan, informed consent, and other information provided to the participants shall be reviewed and approved by the IEC, and the relevant approval documents should be provided to the sponsor.

**Informed consent forms (ICF)**

ICF must be signed before study procedure.

**XVII. Test progress, data saving and summary report**

**1. Test progress**

March, 2011 ~ March, 2013

**2. CRF storage**

Case report form should be reviewed and signed by the principal investigator. After the completion of the test, the case report forms, details of the typical cases and using records of clinical trial drugs should be kept. Researchers should save all the related original data of the subjects, save the related imaging data of CR or PR patients, the original of signed informed consent, CRF copy and drug distribution records.

**3. Final report**

Statistic results are confirmed by the research unit as the basis for writing “short conclusion of clinical trial”. The completed short report is sealed and 1 copy is kept the research unit.

**4. Publication of the experimental results**

Data of this study must be published by the research unit.

**Reference**

1. National Comprehensive Cancer Network. Clinical Practice Guidelines in　Oncology: Breast cancer v.2.2008. http://www.nccn.org/professionals/physician_gls/PDF/breast.pdf. (2008).
2. Bernard-Marty,C., Cardoso,F. & Piccart,M.J. Facts and controversies in systemic　treatment of metastatic breast cancer. Oncologist. 9, 617-632 (2004).
3. Verma,S. & Clemons,M. First-line treatment options for patients with HER-2　negative metastatic breast cancer: the impact of modern adjuvant chemotherapy.　Oncologist. 12, 785-797 (2007).
4. Blum,J.L. et al. Multicenter phase II study of capecitabine in paclitaxel-refractory　metastatic breast cancer. J. Clin. Oncol. 17, 485-493 (1999).
5. Blum,J.L. et al. Multicenter, Phase II study of capecitabine in taxane-pretreated　metastatic breast carcinoma patients. Cancer 92, 1759-1768 (2001).
6. Fumoleau,P. et al. Multicentre, phase II study evaluating capecitabine　onotherapy in patients with anthracycline- and taxane-pretreated metastatic　breast cancer. Eur. J. Cancer 40, 536-542 (2004).
7. O'Shaughnessy,J., Twelves,C. & Aapro,M. Treatment for anthracyclinepretreated　metastatic breast cancer. Oncologist. 7 Suppl 6, 4-12 (2002).
8. O'Shaughnessy,J., et al. Superior Survival With Capecitabine Plus Docetaxel Combination Therapy in Anthracycline-Pretreated Patients With Advanced Breast Cancer: Phase III Trial Results. J Clin Oncol. 20, 2812-2823 (2002).
9. Fumoleau,P. et al. A multicentre phase II study of the efficacy and safety of　docetaxel as first-line treatment of advanced breast cancer: report of the Clinical　Screening Group of the EORTC. Ann. Oncol. 7, 165-171 (1996).
10. Reichardt,P. et al. Multicenter phase II study of oral capecitabine (Xeloda(")) in　patients with metastatic breast cancer relapsing after treatment with a taxanecontaining　therapy. Ann Oncol 14, 1227-1233 (2003).
11. Thomas, ES., et al. Ixabepilone Plus Capecitabine for Metastatic Breast Cancer Progressing After Anthracycline and Taxane Treatment. J Clin Oncol, 25, 1-7 (2007).
12. Miller, KD. et al. Randomized Phase III Trial of Capecitabine Compared With Bevacizumab Plus Capecitabine in Patients With Previously Treated Metastatic Breast Cancer. J Clin Oncol, 23,792-799 (2005).
13. Gelmon,K., Chan,A. & Harbeck,N. The role of capecitabine in first-line treatment　for patients with metastatic breast cancer. Oncologist. 11 Suppl 1, 42-51 (2006).
14. Bajetta,E. et al. Safety and efficacy of two different doses of capecitabine in the　treatment of advanced breast cancer in older women. J. Clin. Oncol. 23, 2155-2161 (2005).
15. 牟希亚等. 绿脓杆菌甘露糖敏感血凝菌毛株. 发明专利，专利号ZL200510059850.X
16. 牟希亚，郭雁群，牟心赤. 菌毛学研究进展. 大连出版社1999.
17. Zhebin Liu, Yifeng Hou, Min Dong, et al. PA-MSHA inhibits proliferation and induces apoptosis through the up-regulation and activation of caspases in the human breast cancer cell lines [J]. J Cell Biochem 2009, 9999:1-12.
18. Zhebin Liu, Yifeng Hou, Jie Zhu, et al. PA-MSHA Inhibits Her-1/EGFR Signaling, Invasiveness and the Metastasis Potential of Breast Cancer Cells both in Vitro and in Vivo Mediated by Type I Pili via a Mannose-Dependent Way [J]. Oncogene. Revision.
19. da Silva Correia J, Ulevitch RJ. MD-2 and TLR4 N-linked glycosylations are important for a functional lipopolysaccharide receptor [J]. J Biol Chem. 2002 Jan 18;277(3):1845-54.
20. 孙文平，付红文，刘妮，吴毓，牟希亚，靳岩，高小平.PA-MSHA菌毛株疫苗对三种癌症患者免疫疗效的观察[J].中华微生物学和免疫学杂志. 2000，20(4): 373-376.
21. 毛启新等.铜绿假单胞菌注射液联合新辅助化疗治疗乳腺癌的临床研究[J]. 中华肿瘤防治杂志. 2010
22. 徐峰，唐中华，李允山等.铜绿假单胞菌制剂在乳腺癌新辅助化疗中的应用[J].中南药学. 2009，7(8): 626-629.
23. 陈小松.紫杉醇联合卡铂每周方案在乳腺癌新辅助化疗中的疗效及安全性研究. 复旦大学硕士学位论文. 2009.
